# Haustoria – arsenals during the interaction between wheat and *Puccinia striiformis* f. sp. *tritici*

**DOI:** 10.1111/mpp.12882

**Published:** 2019-11-27

**Authors:** Qiang Xu, Chunlei Tang, Likun Wang, Congcong Zhao, Zhensheng Kang, Xiaojie Wang

**Affiliations:** ^1^ State Key Laboratory of Crop Stress Biology for Arid Areas and College of Plant Protection Northwest A&F University Yangling Shaanxi 712100 China

**Keywords:** effectors, haustoria, PAMP‐triggered immunity (PTI), *Puccinia striiformis* f. sp. *tritici*

## Abstract

As an obligate parasite, *Puccinia striiformis* f. sp. *tritici* (*Pst*) forms haustoria to obtain nutrients from plant cells for development, and these structures are essential for pathogen survival. To better understand the contribution of haustoria to the interactions with the host plants, we isolated haustoria from susceptible wheat leaves infected with *Pst* race CYR31 and sequenced their transcriptome as well as those of urediospores and germ tubes, and compared the three transcriptomes. A total of 3524 up‐regulated genes were obtained from haustoria, of which 73 genes were related to thiamine biosynthesis, glycolysis and lipid metabolic processes. Silencing seven of the genes reduced the growth and development of *Pst* in wheat. More interestingly, 1197 haustorial secreted proteins (HASPs) were detected in haustoria, accounting for 34% of the total proteins, indicating that these HASPs play important roles in haustorium‐mediated pathogenic progression. Furthermore, 69 HASPs were able to suppress Bax‐triggered programmed cell death in tobacco. Additionally, 46 HASPs significantly reduced callose deposition in wheat using the type III secretion system. This study identified a large number of effectors through transcriptome sequencing, and the results revealed components of metabolic pathways that impact the growth and colonization of the pathogen and indicate essential functions of haustoria in the growth and pathogenicity of *Pst*.

## Introduction

Plant have evolved a multilayered immune system to detect and ward off potential pathogens. One type of conserved molecules, collectively designated pathogen‐associated molecular patterns (PAMPs), is recognized by plant pattern‐recognition receptors (PRRs) on the plant cell membrane, resulting in PAMP‐triggered immunity (PTI), which includes such processes as callose deposition in the plant cell wall and an oxidative burst (Boller and Felix, 2009; Jones and Dangl, 2006). However, to evade or suppress host basal immunity, some invading parasitic fungi invoke a new attack strategy of secreting effectors from haustoria, specialized infection structures that serve as a physiological and pathological bridge between the pathogen and its potential host. Plants respond by deploying a type of nucleotide‐binding site, leucine‐rich repeat proteins to detect pathogen effectors in the host–pathogen co‐evolution cycle (Cui *et al.*, 2015). Once pathogen effectors are recognized, a robust resistance response called effector‐triggered immunity (ETI) ensues together with a hypersensitive response (HR) and accumulation of reactive oxygen species (ROS), followed by systemic resistance to control further colonization by the pathogen (Jones and Dangl, 2006, Wit, 2007). A successful pathogen must elude or undermine this surveillance mechanism and further disarm host multilayer defences (Göhre and Robatzek, 2008). Natural selection helps pathogens evade resistance proteins as a result of loss or diversification of the recognized effectors that suppress ETI (Jones and Dangl, 2006). Because the emergence of new races due to variation and natural selection pressure that removes previously resistant cultivars complicates disease control measures, understanding the plant–pathogen interactions will aid the development and promotion of new strategies for sustained disease control in crops.

Compared with necrotrophic pathogens, the wheat stripe rust pathogen *Puccinia striiformis* f. sp. *tritici* (*Pst*), as an obligately parasitic filamentous fungus, can live only in the host cells where it can form a haustorium, a highly differentiated infection structure that is vital to the development and survival of *Pst* in plant tissues. In addition, many *in planta‐*induced genes are preferentially expressed in haustoria, and their products have been associated with essential metabolic functions and nutrient flux (Hahn and Mendgen, 1997; Jakupovic *et al.*, 2006). Some sugar transporters and amino acid transporters are highly expressed and detected at high concentrations in haustoria for the uptake of sugars and amino acids (Voegele *et al.*, 2001). For example, *HXT1* of *Uromyces fabae* is exclusively expressed in haustoria, is localized exclusively to the haustorial plasma membrane and has substrate specificity for d‐glucose and d‐fructose (Voegele and Mendgen, 2003). Due to the lack of certain typical metabolic genes in the *Pst* genome, such as nitrate/nitrite transporters and nitrate reductases for NH_4_^+^ assimilation, biotrophic fungi must acquire nutrients from their host tissues to promote their own growth and development (Tang *et al.*, 2017). Furthermore, plant cell wall‐degrading enzymes are also induced in *Pst*, and obligate biotrophs, such as the wheat powdery mildew pathogen (*Blumeria graminis* f. sp. *tritici*), may alter plant cell walls to reduce the plant defence response during colonization (Duplessis *et al.*, 2016). Therefore, preferential expression of these genes in haustoria is in agreement with the prominent status of haustoria during plant–pathogen interactions. Indeed, previous studies indicated that haustoria comprise a hub between the pathogen and host for a flood of signal exchange and nutrient uptake, ultimately facilitating pathogen colonization and development (Joel, 2013; Jones and Dangl, 2006).

Overall, haustoria satisfy the needs of biotrophic pathogens, such as acquisition of amino acids and carbohydrates, and function to allow the pathogen to evade host defences (Voegele *et al.*, 2001). Most importantly, as a component of an arsenal, haustoria secrete certain macromolecules, such as effectors, into host tissues to alter the defence balance. A previous study showed that some secreted *B*. *graminis* f. sp. *tritici* effectors target RNA–protein complexes and interfere with RNA‐mediated silencing, thereby contributing to successful infection (Qiao *et al.*, 2013; Spanu, 2015). In addition, effector HaRxL44 in the downy mildew pathogen targets mediator subunit 19a (MED19a) to regulate the balance of jasmonic acid (JA)/ethylene (ET) and salicylic acid (SA) signalling pathways (Caillaud *et al.*, 2013). Regarding effectors in rust fungi, proteome analysis of the wheat leaf rust fungus (*Puccinia triticina*) revealed critical roles in such aspects as energy, general metabolism and gene expression (Song *et al.*, 2011). Moreover, *Pst* effector Pst_02549 accumulates in the plant cell P‐body, a complex structure associated with mRNA decapping, degradation and storage, and this effector may target the P‐body to interfere with RNA metabolism and deregulate host lines of defence (Petre *et al.*, 2016). Thus, pathogen effectors are vital factors that reprogramme the plant immune system to enable colonization.

*Puccinia striiformis* f. sp. *tritici* causes severe damage to wheat production worldwide (Hovmoller *et al.*, 2010). Because haustoria are considered to be an important bridge between the pathogen and host to regulate the microenvironment of their interactions, we screened 3524 differentially expressed genes (DEGs) from haustorial transcripts and selected 73 DEGs related to *Pst* metabolism to determine their expression profiles during the interaction with a susceptible host variety. Using the host‐induced gene silencing (HIGS) assay, seven genes were found to affect the growth and development of *Pst*. In addition, we screened 1197 candidate effectors and found that 69 inhibit programmed cell death (PCD) in *Nicotiana benthamiana* triggered by the pro‐apoptotic protein Bax (Bcl2‐associated X protein). Using a bacterial type III secretion system (T3SS), 46 haustorial secreted proteins (HASPs) were found to suppress PTI in wheat. Overall, the large‐scale functional screening for effectors of obligate biotrophic fungi offers new insight into these important proteins in haustoria and aids in the systematic functional characterization of effectors from haustoria.

## Results

### Identifying transcripts specifically enriched in haustoria

**Figure 1:**
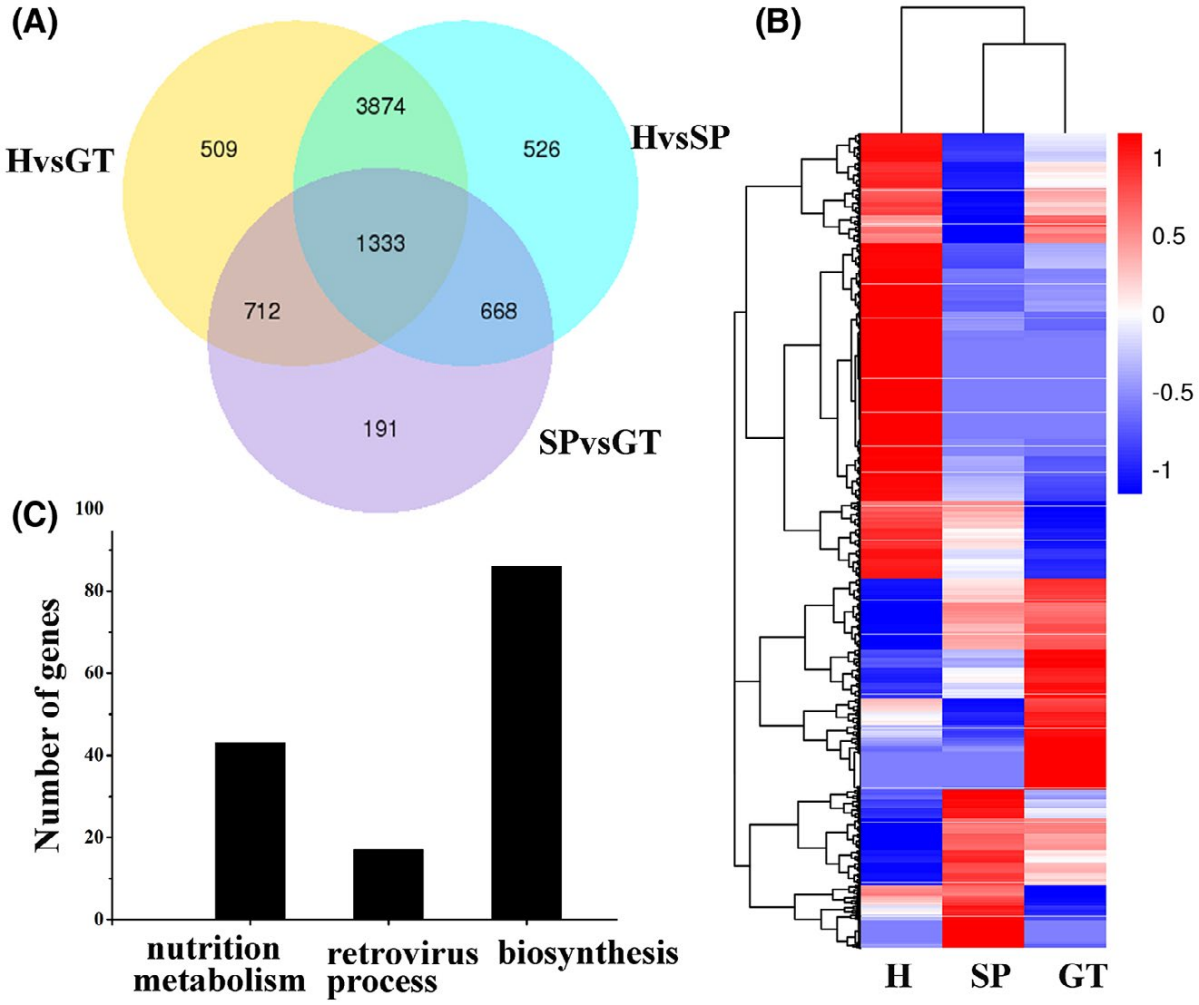
Differentially expressed genes (DEGs) in urediospores, germ tubes and haustoria. (A) Venn diagram of transcript sets showing the number of significant DEGs between urediospores (SP), germ tubes (GT) and haustoria (H). The number of genes was 6428, 6421 and 2924 in the H vs GT, H vs SP and SP vs GT groups, respectively, based on false discovery rate (FDR) < 0.05. (B) Heatmap of DEGs in urediospores, germ tubes and haustoria. The transcript levels are shown with a red‐blue colour scale, where red represents up‐regulation and blue represents down‐regulation. (C) A total of 146 DEGs among these 3524 up‐regulated genes were classified into nutrition metabolism, the retrovirus process, and metabolism and biosynthesis (FDR‐corrected *P*‐values < 0.01).

To further investigate the role of haustoria during *Pst* infection, we isolated haustoria of *Pst* race CYR31 from infected leaves of a susceptible wheat variety using a concanavalin A (Con A) column (Fig. S1), and used RNA sequencing (RNA‐Seq) analysis of urediospores, germ tube and haustorial tissues to identify haustorial expression of transcripts. After filtering small reads, the number of reads for the urediospores (SP), germ tube (GT) and haustorium (H) reached 31, 23 and 14 million, accounting for 83.2%, 86.8% and 15.0% of the mapping rate, respectively (Table S1). We also conducted an analysis of altered expression after normalization. Based on the fragment per kilobase per million mapped reads (FPKM) as the expression level, 7813 DEGs based on RNA‐Seq of urediospores, germ tubes and haustoria were selected using a false discovery rate (FDR) < 0.05 (Fig. 1A). To focus on DEGs in haustoria, there were approximately 5776 (4339 up‐ and 1437 down‐regulated) and 5878 (4183 up‐ and 1695 down‐regulated) DEGs compared with the urediospores and germ tubes, respectively, when using the parameters of |log_2_ (fold change)| > 1 and FDR < 0.05 (Fig. 1B, Tables S2 and S3). To determine the biological function of haustoria in the wheat–*Pst* interaction, we selected target DEGs that were up‐regulated in haustoria and obtained 3524 DEGs with a fold change >2 compared with those in the urediospores and germ tubes (Table S4). We assigned priority to these DEGs and categorized them into biological processes, cellular processes and molecular processes using Blast2GO. As shown in Fig. 1C, based on the condition of FDR < 0.01 and removing DEGs without functional annotation, 146 DEGs out of the 3524 up‐regulated genes were classified, with 43 related to nutrition metabolism, 17 to the retrovirus process and 86 to metabolism and biosynthesis (Fig. 1C and Table S5). Some of the up‐regulated genes were found to be related to thiamine biosynthesis, glycolysis and lipid metabolic processes. Two genes, *Pst_16188* and *Pst_19493*, were annotated to participate in the biosynthesis of carbohydrates, amino acids and thiamine (Table S5). *Pst_11012* was predicted to encode an aspartic protease that may contribute predominantly to proteolytic activities. *Pst_12394* was found to possibly encode a glucose‐6‐phosphate 1‐epimerase involved in lactose assimilation and cellulose formation. In general, the annotations indicated that many of the genes may be involved in the primary metabolism and physiology of haustoria.

### The expression patterns of 73 DEGs during the wheat–*Pst* interaction

**Figure 2:**
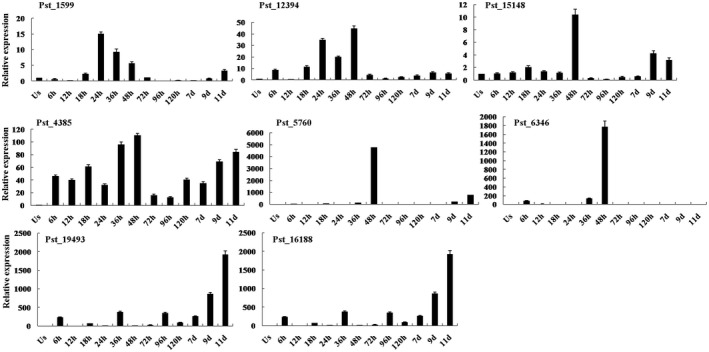
Analysis of transcript level patterns of eight metabolism‐related genes by RT‐qPCR. Abundant transcripts were from urediospores and other stages of *Puccinia striiformis* f. sp. *tritici* (*Pst*) infection. Us, urediospores of *Pst*, 6, 12, 24, 36, 48, 72, 96 and 120 hours post‐inoculation of wheat leaves, and 7, 9 and 11 days post‐inoculation of wheat leaves. The standard error was obtained from three independent replicates.

To determine the transcript levels of DEGs during the process of *Pst* infection, we examined corresponding transcript levels at different *Pst* stages, including the urediospores, germ tubes and other important infection stages. Randomized 73 highly expressed genes from the 146 DEGs were successfully evaluated for expression at these stages by quantitative reverse‐transcription PCR (RT‐qPCR), including 20 genes specifically induced in the urediospores, seven genes specifically induced in germ tubes and 46 up‐regulated genes in haustoria (Figs 2 and S6). For example, *Pst_12394* was significantly up‐regulated at 18 h post‐inoculation (hpi), with a peak at 48 hpi. In general, 18–48 hpi is considered an important stage for infectious hypha and haustorium formation, and this early stage of infection is critical for pathogen colonization (Cheng *et al.*, 2015). Expressions of *Pst_16188* and *Pst_19493* were up‐regulated from urediospore germination, peaking at 256 hpi (Fig. 2).

### Seven metabolism‐related genes reduce the growth of *Pst* in wheat

**Figure 3:**
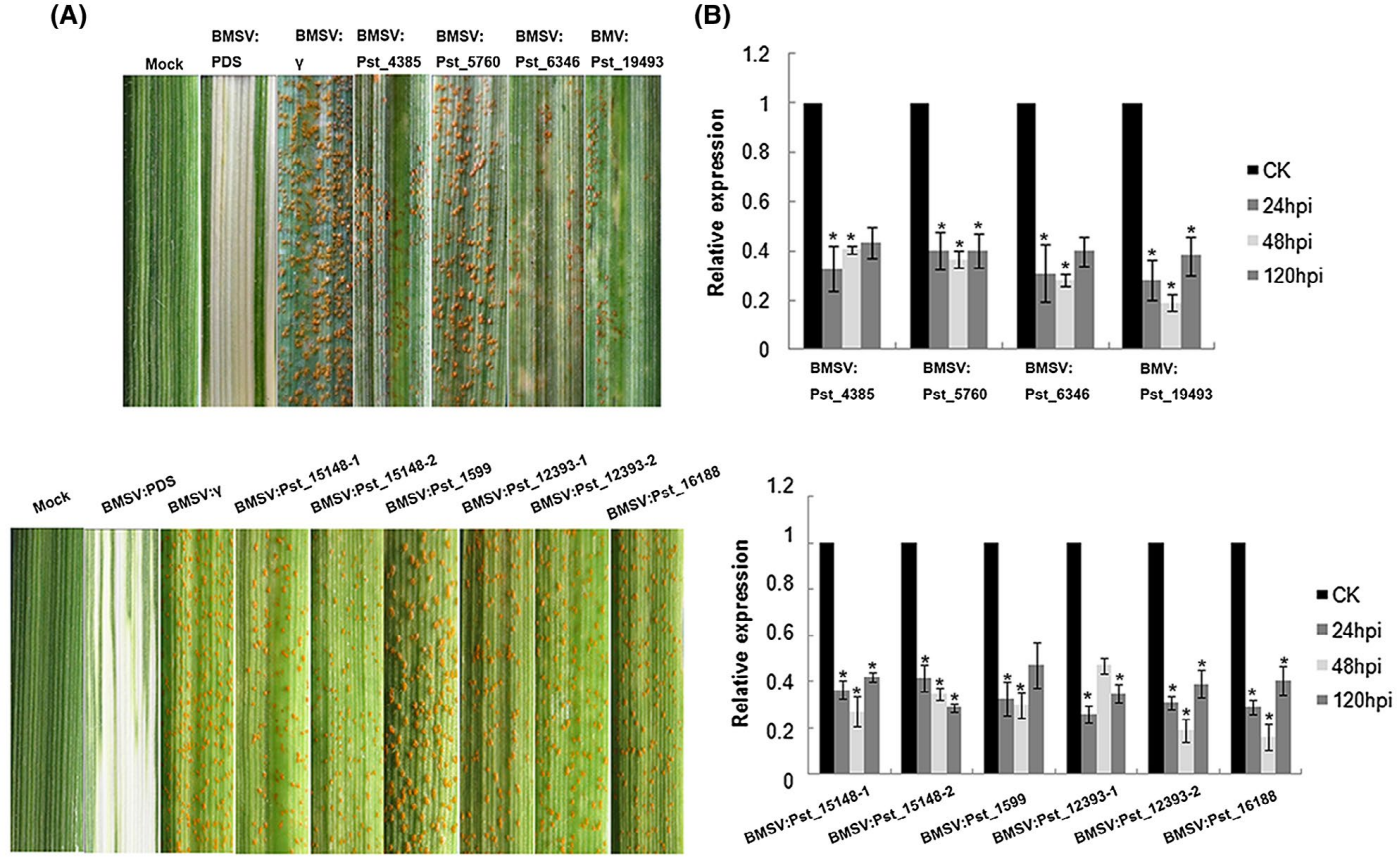
Functional analysis of eight metabolism‐related genes of *Puccinia striiformis* f. sp. *tritici* (*Pst*) by host‐induced gene silencing. (A) The fourth leaves inoculated with urediospores of *Pst* race CYR31 were photographed at 15 days post‐inoculation after silencing. Mock, wheat leaves treated with FES buffer alone. Mild chlorotic mosaic symptoms were observed in wheat inoculated with BSMV:*TaPDS* as a control. (B) The silencing efficiency of metabolism‐related genes in silenced plants. The relative expression of these genes was calculated by the 2^−ΔΔCt^ method. The control (CK) data were from wheat leaves infected with the empty vector (BSMV:γ) at 24, 48 and 120 hours post‐inoculation (hpi). Bars represent mean values ± standard error of three independent sample collections.

A stable and efficient transformation system is not available for *Pst*. Nonetheless, virulence genes and pathogenicity mechanisms can be explored using transient silencing via barley stripe mosaic virus (BSMV)‐mediated host‐induced gene silencing (HIGS) (Nowara *et al.*, 2010). Therefore, from the 46 up‐regulated genes in the haustorial stage identified in the RT‐qPCR experiment, we randomly selected 14 DEGs that were more highly expressed in these stages than in urediospores and germ tubes. We used the HIGS approach to determine the possible functions of the 14 genes (Table S8). Mild chlorotic mosaic symptoms were observed as the virus‐infected phenotype, and positive control *TaPDS*‐knockdown plants exhibited photobleaching on the fourth leaves (Fig. 3A). Silencing of *Pst_11012*, *Pst_22758*, *Pst_12394*, *Pst_16188*, *Pst_6346*, *Pst_15148* and *Pst_19493* obviously changed the gene expression level, whereby the knockdowns of these genes were not beneficial to hyphal growth in plants (Figs 3A and S2A). To accurately measure *Pst* hyphal development in the knockdown plants, histological observations revealed that, compared with the control, the length of hyphae decreased significantly at 48 hpi and growth was significantly reduced at 120 hpi in the *Pst* gene‐knockdown plants (Table S9 and Fig. S3). To confirm the efficiency of silencing, target gene transcript levels in the knockdown plants were analysed using RT‐qPCR. The abundance of these transcripts decreased to 20–40% compared with the control plants (Figs 3B and S2B), demonstrating successful silencing of these target genes. These results indicate that the growth of *Pst* is compromised after silencing of these genes. Nevertheless, the pathogenic mechanisms and crosstalk between the host and pathogen remain unclear, although the present results indicate that these metabolism‐related genes are associated with the growth and development of *Pst*.

### Identification of *Pst* effectors using RNA‐Seq

**Figure 4:**
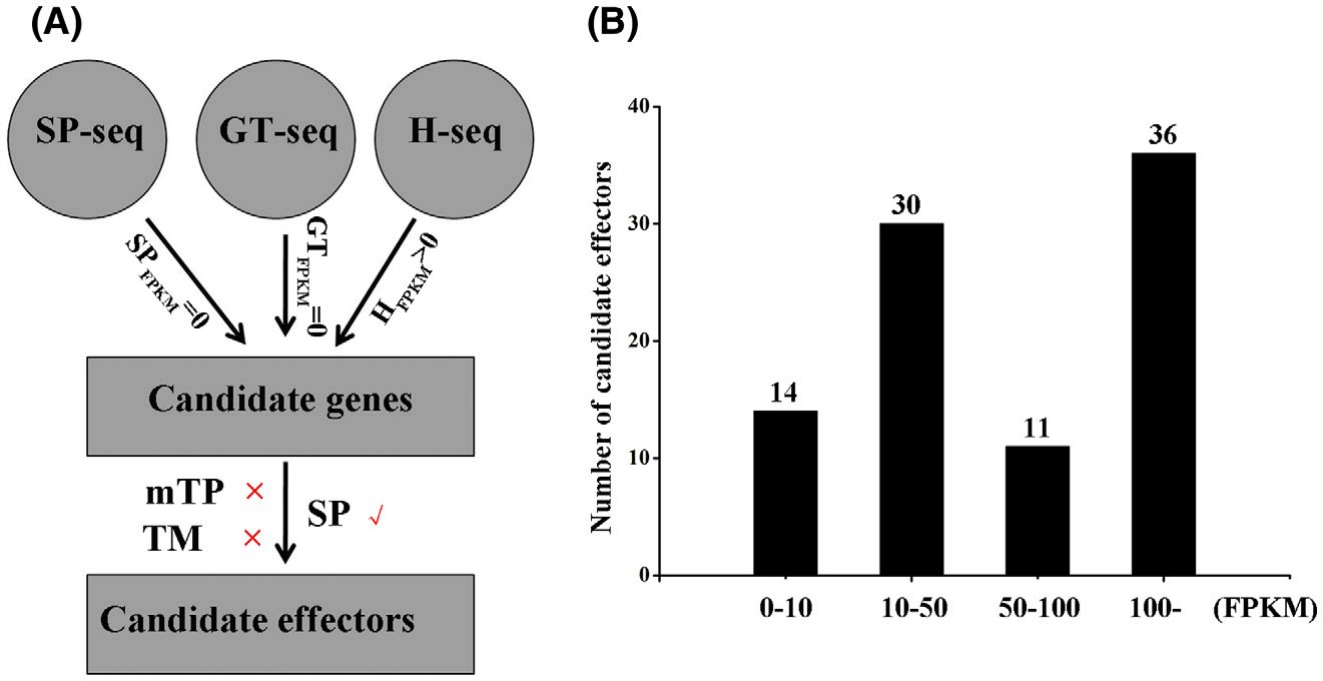
Candidate effectors for the urediospores (SP‐seq), germ tubes (GT‐seq) and haustoria (H‐seq) were selected. (A) The pipeline for predicted haustorial secreted proteins (HASPs). Signal peptide (SP), mitochondrial targeting signals (mTP) and transmembrane domains (TM) were predicted using SignalP v. 3.0 with cutoffs > 0.5, TargetP (v. 1.1) with cutoffs > 0.5 and TMHMM (v. 2.0) with cutoffs > 0.5, respectively. (B) The distribution of the transcript levels of 91 effectors in this study. HASPs were distributed in four categories (0–10, 10–50, 50–100 and 100– FPKM) according to the different expression levels.

Because pathogen effectors likely disturb the host plant immunity, we searched the RNA‐Seq datasets of haustoria, urediospores and germ tubes for secreted proteins containing a predicted signal peptide (SP) using SignalP v. 3.0 (Bendtsen *et al.*, 2004; Saunders *et al.*, 2012), and then selected from the 3524 DEGs for those specifically expressed in haustoria but not in urediospores or germ tubes. After removal of secreted proteins containing mitochondrial targeting signals and transmembrane domains, 1197 candidate effectors genes were identified (Fig. 4A, Tables S6 and S7). Because these effector genes were specifically expressed in haustoria, they were temporarily designated as HASPs. To screen HASPs and determine their functions in the wheat–*Pst* interaction, we divided them into four categories (0–10, 10–50, 50–100 and 100– FPKM) according to the different expression levels of HASPs. We randomly selected 91 HASPs with different expression levels for further experiments, including 36 with expression levels FPKM > 100, 11 with FPKM between 50 and 100, 36 with FPKM between 10 and 50) and 14 with FPKM < 10 (Fig. 4B).

### HASPs suppress Bax‐triggered PCD

**Figure 5:**
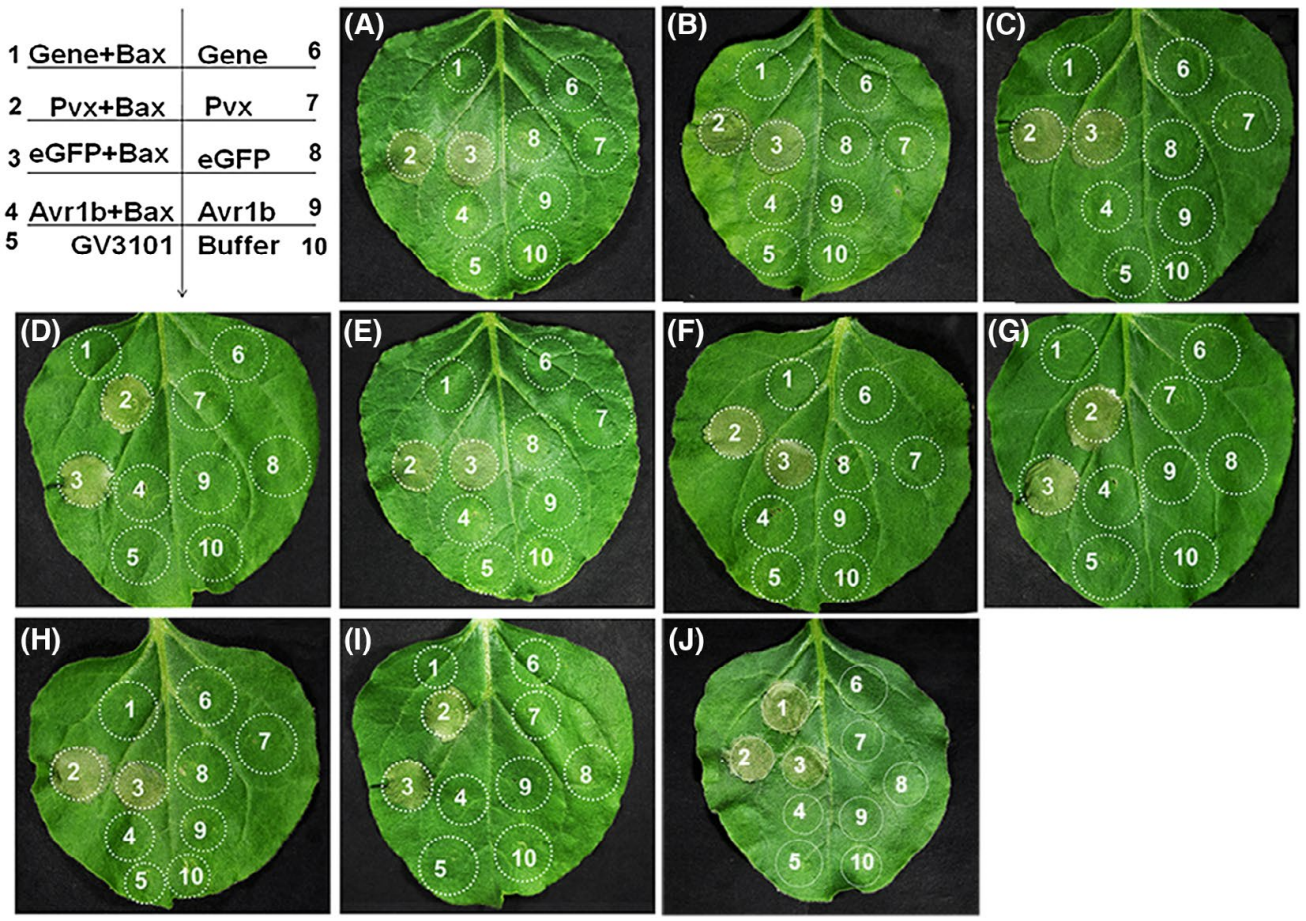
Suppression of programmed cell death by *Puccinia striiformis* f. sp. *tritici* (*Pst*) haustorial secreted proteins (HASPs) in *Nicotiana benthamiana*. Leaves were infiltrated with buffer, empty *Agrobacterium tumefaciens* cells or *A. tumefaciens* cells containing a vector carrying HASP candidates, a negative control gene (eGFP), empty vector or a positive control (Avr1b); 24 h later, *A*. *tumefaciens* cells carrying Bax were infiltrated. Here, we show only representative results at 6 days after infiltrating Bax. The top left panel is the injection layout. A–J: HASP219, HASP236, HASP248, HASP260, HASP256, HASP275, HASP241, HASP264, HASP272 and HASP251.

Previous studies indicated that suppressing defence‐related PCD may contribute to pathogen virulence (Dou *et al*., 2008b), and that *N*. *benthamiana* is a model plant for illuminating effector functions in obligate biotrophic pathogens (Armstrong *et al.*, 2005; Petre *et al.*, 2016; Wang *et al.*, 2011). Thus, we screened the 91 selected HASPs using an *Agrobacterium tumefaciens‐*mediated transient expression assay in *N*. *benthamiana* (Table S10). *Agrobacterium tumefaciens* GV3101 constructs containing each effector gene, eGFP (a negative control), Avr1b (a positive control) and pGR106 (a blank control) were infiltrated into leaves at appropriate concentrations. As shown in Fig. 5, Avr1b suppressed PCD and 69 of the selected HASPs also suppressed Bax‐triggered PCD when infiltrated 24 h prior to Bax (Fig. 5). However, the remaining 22 HASPs did not trigger PCD, including HASP251 shown in Fig. 5 as an example. To confirm these results, various control proteins were expressed in leaves, followed by isolation after 72 h. As revealed by western blotting, Bax and eGFP proteins were detected as expected (Fig. S4). These results demonstrate that 69 HASPs were able to suppress Bax‐triggered PCD, warranting further investigation of their functions.

### HASPs suppress wheat PTI induced by the T3SS

**Figure 6:**
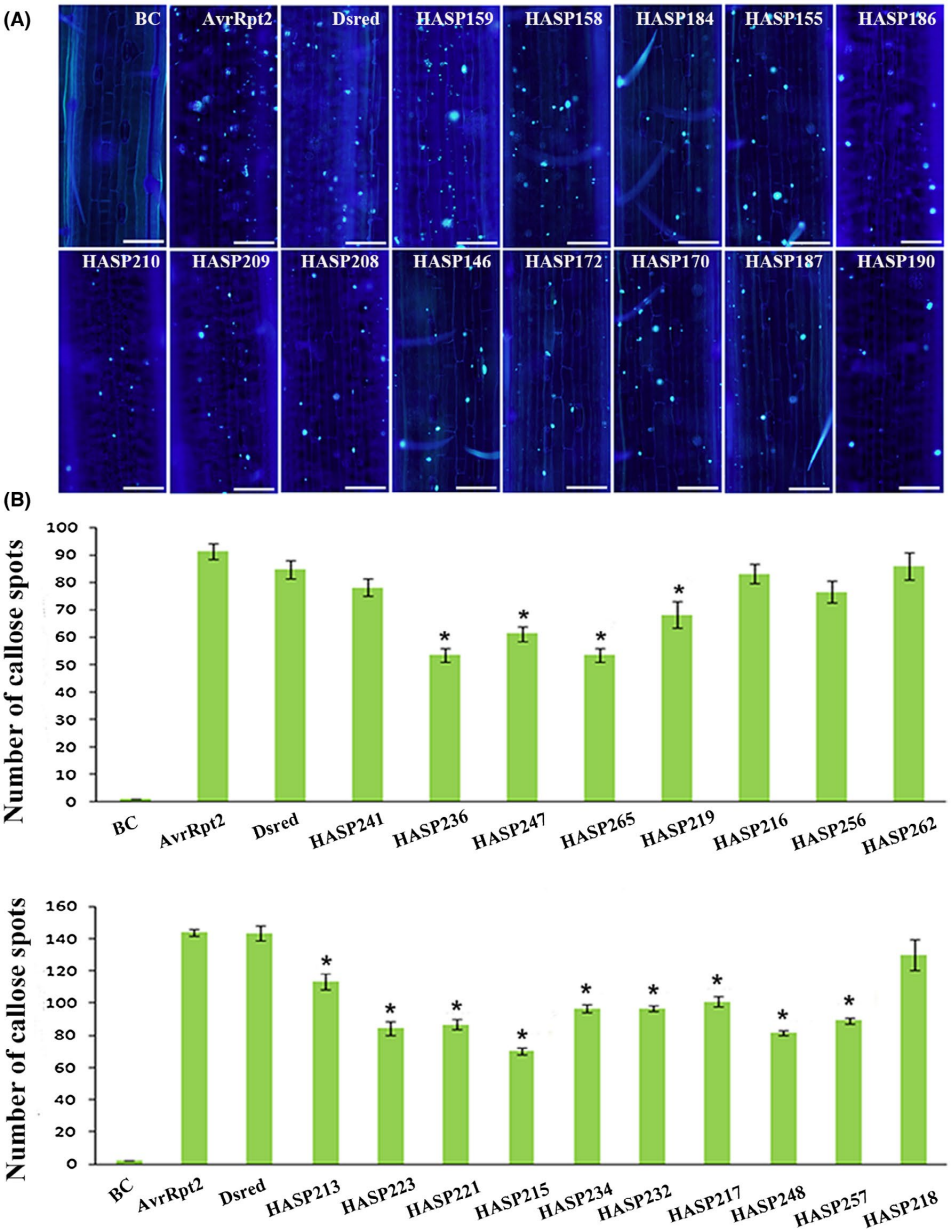
Haustorial secreted proteins (HASPs) suppress host PAMP‐triggered immunity (PTI) via the bacterial type III secretion system. (A). Representative wheat leaves stained with aniline blue for callose deposition (bar = 100 μm). The BC sample shows a plant inoculated with water. As a negative control, *Pseudomonas fluorescens* strain EtHAn carrying Dsred was incapable of suppressing callose deposition. Representative pictures obtained at 24 h after inoculation and aniline blue staining and observed at ×10 magnification with an Olympus BX‐51 microscope are shown. (B) The average number of callose foci per field of view (1 mm^2^) was calculated from 30 fields. The BC sample is plant inoculated with water and Dsred as a negative control. The average and standard errors were from three independent samples. Significance was assessed by Student's *t*‐test (asterisk, *P* < 0.05).

Plants launch a primary defence consisting of various responses, such as callose deposition, which is triggered by PTI during pathogen infection (Boller and Felix, 2009; Jones and Dangl, 2006); thus, measuring callose deposition is widely used for assessing PTI (Hauck *et al.*, 2003; Xin and He, 2013). Accordingly, we selected 69 HASPs that could suppress Bax‐triggered PCD for further analysis of their ability to suppress PTI in wheat leaves. Constructs using pEDV6 and 68 of these HASPs were generated and transformed into the *Pseudomonas fluorescens* strain Effector to Host Analyzer (EtHAn), which delivers foreign effectors into wheat (Thomas *et al.*, 2009). As HASP253 was not successfully produced, it was not included in this experiment. At 24 h after infiltration, the control, EtHAn containing Dsred or AvrRpt2, did not block the PTI response in wheat, and the blank control, plants infiltrated with MgCl_2_ buffer, exhibited no callose accumulation (Figs 6 and S5). In total, 46 HASP candidates reduced callose deposition induced by *P. fluorescens* EtHAn in wheat cv. MX169 (Figs 6 and S5). However, 22 of the 46 HASPs, including HASP262 (Figs 6 and S5), failed to suppress callose deposition, resulting in suppression of PTI. Based on the suppression of callose deposition, the 22 HASPs can be regarded as candidate effectors that may inhibit plant PTI. However, their specific functions during the wheat–*Pst* interaction should be further investigated.

## Discussion

*Puccinia striiformis* f. sp. *tritici* (*Pst*) deprives nutrients from wheat during infection, resulting in the loss of carbohydrate accumulation during wheat development and ultimately reducing wheat yield (Hovmoller *et al.*, 2010). As one of the hallmarks of obligate biotrophic fungi, haustoria penetrate only the plant cell wall and expand inside that cell to form a bridge between the pathogen and host, instead of killing the plant cell at the front of the mycelium (Catanzariti *et al.*, 2006). The formation of haustoria facilitates the uptake of nutrients, including amino acids and carbohydrates, from the host cell for rust fungus colonization and development (Daly *et al.*, 1967; Eisenreich *et al.*, 2013). Without a doubt, haustoria are of vital importance to establish the parasitism between the fungal pathogens and plant. Therefore, studying the function of haustoria will greatly enhance the understanding of the *Pst* pathogenesis mechanism. Transcriptome sequencing of haustoria, germinated spores and urediospores can provide functional genomics data for better understanding of the metabolic functions of infection structures and prioritizing candidate effector genes for further functional studies of this obligate biotrophic fungus.

In a previous study, haustoria of *Pst* were isolated using a Percoll gradient method and 4485 transcripts were annotated (Garnica *et al.*, 2013). These authors identified many genes related to fungal development and metabolism such as cell wall modification enzymes, sugar transporters and thiamine biosynthesis. From the 437 predicted secreted proteins, 295 were found to be overexpressed in haustoria through digital expression analysis (Garnica *et al.*, 2013). That study provided a comprehensive view of gene expression in germinated urediospores and haustorial stages. In our study, we analysed the transcriptome of the urediospores, germ tubes and haustoria, and obtained 3524 genes that were up‐regulated in haustoria compared with urediospores and germ tubes, including 1197 HASPs. Most importantly, we also determined the function of several HASPs during infection of wheat by using the T3SS. Our results of the functional categories revealed by gene annotation and pathway analysis also provide a better understanding for how some DEGs function in the development of haustoria and in the interaction between host and pathogen. Previous studies have revealed some highly expressed genes in haustoria. For example, Jakupovic *et al.* (2006) identified much higher expression of *in planta*‐induced genes, which are involved in the metabolic processes, in haustoria than other structures or stages of the bean rust fungus, *U. fabae*. *Pst_22758*, which is a ubiquitous heat shock protein containing a DnaJ domain, may be involved in protein folding, unfolding, transport and degradation in haustoria (Duppre *et al.*, 2011; Qiu *et al.*, 2006). *Pst_27050* may be involved in the glycometabolism with a raffinose alpha‐galactosidase activity. In the present study, some DEGs were highly induced at different infection stages, suggesting spatiotemporal regulation during the infection process. In contrast, several DEGs, for example H‐2597, had a low expression level at the infection process in the RT‐qPCR experiment, which were different from the results of RNA‐Seq (Fig. S6). However, RNA‐Seq is used to obtain global gene expression from computational predictions, and it may be less effective with low coverage genes. Conversely, RT‐qPCR analyses generate relatively high expression values compared with the result of RNA‐Seq (Roberts *et al.*, 2011). In general, subtle differences are often found between RT‐qPCR and RNA‐Seq due to the different measurements and different normalization processes. Therefore, the expression levels of individual genes from RNA‐Seq or microarrays often need to be further confirmed by RT‐qPCR.

Host‐induced gene silencing is an effective approach to elucidate the function of genes of biotrophic pathogens (Nowara *et al.*, 2010). As *in planta*‐induced genes are often involved in biosynthetic activities, we silenced 14 metabolism‐related genes that were predominantly or highly expressed in haustoria and found that seven genes influenced the growth and development of *Pst* in silenced plants, indicating that these genes are involved in infection. Furthermore, the seven genes were predicted to be involved in cell growth, differentiation and the biosynthesis of carbohydrates, amino acids and thiamine. Silencing *Pst_22758* decreased *Pst* pathogenicity and influenced the growth of hyphae (Table S9), indicating that *Pst_22758* play a positive role in fungal development during the plant–*Pst* interaction. It is interesting that *Pst_12394* encodes a glucose‐6‐phosphate 1‐epimerase involved in glycolysis. Obligate biotrophic fungi absorb and utilize host sugars via haustoria for their growth and development. Some sugar transporters and amino acid transporters on the membrane of haustoria function by facilitating acquisition of nutrients from the host. The HXT1p sugar transporter, encoded by the *HXT1* gene*,* which showed the highest transcript level in haustoria, transports glucose and fructose via a plasma membrane H^+^‐ATPase (Voegele *et al.*, 2001). The glucose and fructose absorbed enter pathogen metabolic pathways to provide energy. Glucose‐6‐phosphate 1‐epimerase plays a major role in transforming glucose‐6‐phosphate into fructose‐6‐phosphate, providing ATP and NADPH to *Pst*, in agreement with the observation that silencing *Pst_12394* decreased *Pst* expansion in silenced plants. We also found that *Pst_16188*, which is involved in thiamine biosynthesis and related to metallothioneins, was highly induced at 36 hpi, 9 days post‐inoculation (dpi) and 11 dpi. Thiamine is a cofactor necessary for many metabolic enzymes, is able to induce expression of resistance genes and is involved in the H_2_O_2_ pathway in plants (Boubakri *et al.*, 2012). Previous studies have reported that all haustorium‐forming pathogens obtain thiamine from the host because of the lack of a thiamine biosynthetic pathway, indicating that thiamine has an irreplaceable role in haustorium metabolism and hyphal development (Kemen *et al.*, 2011). In addition, metallothioneins are associated with scavenging of reactive oxygen radicals (Ruttkaynedecky *et al.*, 2013). Thus, knocking down *Pst_16188* may result in obstructing thiamine uptake from the host and thiamine metabolism in haustoria, which is consistent with the features of obligate biotrophic fungi. Overall, haustoria are irreplaceable structures in metabolism and mediate extensive nutrient trafficking between wheat and rust fungi. Collectively, transcriptomic analysis of haustoria helps to identify genes that are preferentially expressed in haustoria and related to interactions with living host tissues.

As an arsenal for invasion, haustoria are responsible for the production and secretion of virulence factors, termed effectors, which have versatile roles in the physiological and immune responses of host cells. Effector HopAI1 from *Pseudomonas syringae*, for instance, dephosphorylates host MAPKs to alter signalling in host tissues, resulting in decreased immune function (Zhang *et al.*, 2007). In the present study, 1197 candidate effectors were identified based on the haustorial transcriptome and bioinformatic results. Due to the lack of PFAM domains in effectors and a transformation system for *Pst*, it is difficult to study gene functions. However, *N. benthamiana* provides a model for establishing a high‐throughput pipeline for effectors of biotrophic pathogens (Petre *et al.*, 2016; Wang *et al.*, 2011). Based on transcriptomics and large‐scale functional screening, 69 HASPs suppressed Bax‐triggered PCD when infiltrated into tobacco 24 h prior to Bax (Fig. 5). Bax‐triggered PCD is similar to HR, and suppressing Bax‐triggered PCD is considered a feasible method for primary screening of pathogen effectors in non‐host model plants (Abramovitch *et al.*, 2003; Dou *et al.*, 2008). The fact that HASPs suppress Bax‐triggered PCD indicates that some *Pst* effectors have the potential to disturb plant immunity. Furthermore, we suggest that a T3SS might explain the ability of the pathogen to evade host surveillance. In total, 46 HASPs decreased callose deposition induced by bacteria, indicating that these candidates can overcome the first line of plant defence (Fig. 6). T3SSs generally apply when studying the function of pathogen effectors and the plant–pathogen interaction. The ability of pathogens to suppress PTI as a key virulence strategy is a priority for screening virulence effectors (Fabro *et al.*, 2011).

In summary, *Pst* haustoria are not only regarded as means to acquire nutrients for *Pst* growth but are also regarded as an arsenal of effectors to evade the host surveillance system and undermine host multilayer defences. The study of the metabolic processes in haustoria provides important clues for the growth and development of biotrophic fungi. Due to the critical role of effectors, it will be essential to conduct research on their function during interaction between the host and pathogen. This study provides a foundation for future studies on the function of haustoria and effectors, understanding pathogenesis mechanisms and increasing the ability to devise effective strategies to control diseases caused by pathogenic rust fungi.

## Experimental Procedures

### Isolating haustoria from infected wheat leaves

Haustoria were purified using the Con A adsorption column chromatography method described previously (Garnica *et al.*, 2013). Approximately 20 g of infected wheat leaves at 7 dpi were gently washed, placed in 160 mL of homogenization buffer (20 mM MOPS pH 7.2, 300 mM d‐sorbitol, 0.1% bovine serum albumen (BSA), 0.2% PEG6000, 0.2% β‐mercaptoethanol) and homogenized for 20 s at the maximum speed (14 000 rpm) in a blender. The suspension was filtered twice through 20‐µm nylon membranes into 50‐mL centrifuge tubes and centrifuged at 2740 *g* for 5 min at 4 °C. The supernatant was discarded, and 2 mL of suspension buffer (10 mM MOPS, 300 mM d‐sorbitol, 1 mM CaC1_2_, 0.2% BSA, 1 mM KCl, 1 mM MgC1_2_, pH 7.2) was added. The suspension was slowly loaded onto a Sepharose 6 MB coupled to a Con A column, which was incubated at 4 °C for 15 min and then washed three times with the suspension buffer. Haustoria were eluted from the columns by agitation with a wide bore sterile pipette three times. The eluate was transferred into a prechilled 1.5‐mL tube, centrifuged at 22 500 *g* at 4 °C for 5 min and the supernatant carefully discarded. The pellet was quickly frozen in liquid nitrogen and stored at −80 °C for RNA extraction.

### RNA sample preparation, sequencing and analysis

For RNA sample preparation, urediospores of *Pst* race CYR31 were harvested from infected wheat leaves at 14 dpi and stored at −80 °C for RNA extraction. A germ tube mat was obtained from urediospores floating uniformly on the surface of water in a glass dish at 9–12 °C overnight for germination. The mat of intertwined germ tubes was collected carefully, blotted off water on a 3‐M filter paper and stored at −80 °C for RNA extraction. Frozen haustoria, germ tube mat, urediospores and infected leaves were ground in liquid nitrogen, and RNA was isolated with the TRIzol reagent (Invitrogen, Carlsbad, CA, USA) following the manufacturer’s instructions. Approximately 5–10 μg of total RNA from isolated haustoria, germ tubes or urediospores was sequenced using an Illumina HiSeq system by Shbio RNA‐Seq Biotechnology (Shanghai, China). Barcode trimming, quality filtering and adapter removal were carried out with software FastX (v. 0.0.13). Terminal nucleotides at the 3′ end with sequencing quality below Q10 and reads <20 nucleotides were discarded. Then, all high‐quality reads were assembled using the trinity platform to reconstruct a unigene library. DEG analysis was performed with the Bioconductor package DESeq (|log_2_(FoldChange)| > 1 and *q* < 0.05). To reveal gene regulatory networks on the basis of molecular functions, biological processes and cellular components, the transcripts differentially expressed in urediospores, germ tubes and haustoria were analysed through BLAST2GO. GO terms and KEGG pathways with FDR‐corrected *P*‐values < 0.05 were considered statistically significant.

### Bacterial strains, plants and growth conditions

Bacterial strains included *A*. *tumefaciens* GV3101 for transient expression in *N*. *benthamiana*, *Escherichia coli* DH5α and JM109 for bacterial transformation, and *P. fluorescens* EtHAn for delivering secreting effectors into wheat leaves. *Nicotiana* plants were grown in a growth chamber at 25 °C with a light regime of 16 h:8 h light:darkness. Wheat cultivar Suwon 11, MX169 and *Pst* race CYR31 from our laboratory were used in this study. CYR31 urediospores were produced on Suwon 11 in a greenhouse at 16 °C as previously described (Kang *et al.*, 2002). Briefly, a total of 20 mg of fresh urediospores was suspended in 2 mL water and then used to inoculate leaves of wheat seedlings at the 2‐leaf stage using a fine paintbrush. After inoculation, seedlings were kept in a humid chamber for 24 h at 15 °C and then were returned to the growth chamber.

### RT‐qPCR analysis

For RT‐qPCR analysis, the second leaves of Suwon 11 infected with CYR31 collected at 6, 12, 18, 24, 36, 48, 72 and 120 hpi, and 7, 9 and 11 dpi, as well as frozen germ tubes and urediospores were used to extract total RNA as described above. First‐strand cDNA was synthesized using the GoScript reverse transcription system (Promega Corp., Madison, WI, USA). To detect the expression patterns, the transcript levels of 74 DEGs were measured through RT‐qPCR with RNA isolated from urediospores of *Pst* CYR31 and infected leaves at 6, 12, 18, 24, 36, 48, 72, 120 hpi, and at 7, 9 and 11 dpi. The transcript levels of 14 metabolism‐related genes in silenced plants infected with CYR31 were assayed through RT‐qPCR with RNA samples isolated from infected leaves at 24, 48 and 168 hpi after silencing. RT‐qPCR was carried out with a 7500 Real‐Time PCR System (Applied Biosystems, Foster City, CA, USA) under the following conditions: 95 °C for 10 min; 40 cycles of 95 °C for 15 s and 55 °C for 20 s; followed by 95 °C for 15 s and 60 °C for 1 min and 95 °C for 15 s to obtain melting curves. The relative expression of these genes was measured using the comparative 2^−ΔΔCt^ method. *Pst_EF* and *Ta_EF* were used as internal reference genes.

### Construction of plasmids

For the HIGS assay, the specific fragments used for gene silencing were acquired from cDNA of Suwon 11 infected with CYR31. The PCR products were ligated into the BSMV:γ vector after digestion with the restriction enzymes *Not*I and *Pac*I (Takara, Japan). For pGR106, we amplified HASP candidate genes from the cDNA of Suwon 11 infected with CYR31, digested them with enzymes *Cla*I, *Sal*I or *Smal*I, and cloned into pGR106. For pEDV6, the coding sequence (without a signal peptide) was cloned and recombined in pDONR221 following the instructions of the Gateway BP clonase II enzyme mix and Gateway LR clonase enzyme mix II (Invitrogen, Carlsbad, CA, USA). All plasmids in this study were validated by Beijing AuGCT DNA‐SYN Biotechnology (Beijing, China). The primers used in this study are listed in Table S11.

### Transient protein expression in *N. benthamiana*

The recombinant plasmids were introduced into *A*. *tumefaciens* GV3101 cells by electroporation and grown on Luria‐Bertani (LB) plates with kanamycin (50 mg/L) and rifampicin (40 mg/L). Transformants were identifed by PCR. The recombinant bacteria were cultured in LB medium with kanamycin (50 mg/L) and rifampicin (40 mg/L), harvested and washed three times with 10 mM MgCl_2_. The cell suspension was diluted to an OD_600_ of 0.4, incubated at room temperature for 3 h, and infiltrated into 4‐ to 6‐week‐old *N. benthamiana* plants. Bacteria containing HASP genes were injected into leaves prior to infiltration of Bax‐carrying cells. Leaves were harvested at 72 h post‐injection, frozen in liquid nitrogen and stored at −80 °C for protein extraction. Protein extracts were prepared through trichloroacetic acid (TCA)‐acetone precipitation. First, ground leaf tissue was suspended in ten times the volume of 10% TCA‐acetone solution and stored overnight at −20 °C. The solution was centrifuged for 20 min at 4 °C, and the precipitate was washed two times with ten times the volume of acetone solution. The vacuum‐dried precipitate was dissolved in protein extraction buffer (8 M urea, 4% 3‐[(3‐Cholamidopropyl)dimethylammonio]propanesulfonate (CHAPS), 0.5% ampholyte pH 3.5–9.5, 40 mM Tris‐base). Western blotting was performed using anti‐Bax monoclonal antibodies combined with a secondary goat anti‐mouse IgG (whole molecule)‐peroxidase conjugate (Sigma‐Aldrich, Shanghai, China), with enhanced Chemiluminescent Peroxidase Substrate (Thermo Scientific, Rockford, IL, USA) for 5 min, followed by exposure for 60 s up to several minutes depending on the signal strength.

### PTI suppression assays

For suppression of PTI in wheat cultivar MX169, constructs were transformed into *P. fluorescens* EtHAn and cultured overnight on King’s B medium with gentamicin (50 mg/L) and chloromycetin (25 mg/L) at 28 °C. Bacterial cultures were washed and adjusted to OD_600_ = 1.0 with 10 mM MgCl_2_ buffer. The bacteria were infiltrated into the second leaves with a needleless syringe. PTI was allowed to develop at 25 °C, and the leaves were further harvested at 24 hpi for measurement of callose deposition, which involved clearing and staining with aniline blue at 24 h after infiltrating the effectors. In brief, the leaf fragments were decolorized and washed twice with 50% (v/v) ethanol for 15 min, rinsed with water and stained with aniline blue solution (67 mM K_2_HPO_4_, 0.05% (w/v) aniline blue). The degree of callose deposition was determined in fields of 1 mm^2^ with a BX‐51 microscope (Olympus Corp., Tokyo, Japan). For each sample, 30 fields were measured in each biological replicate.

### BSMV‐mediated gene silencing

Special fragments were used to silence the target genes (Table S11). Plasmids containing BSMV α, β and recombinant vector were digested by restriction enzymes *Mlu*I, *Spe*I and *Bss*HII for linearization, respectively, and then 14 gene fragments were transcribed into RNA *in vitro*. The RNA was diluted five times for inoculation; the RNA of each target gene, α and β genes, and FES buffer were mixed at a ratio of 1:1:1:25, as previously described (Holzberg *et al.*, 2002). FES buffer consisted of 0.1 M glycine, 0.06 M K_2_HPO_4_, 1% w/v tetrasodium pyrophosphate, 1% w/v bentonite and 1% w/v celite (pH 8.5). The mixture was inoculated individually onto the second leaves of wheat seedlings as described previously (Hein *et al.*, 2005). The inoculated wheat leaves were maintained at 25 °C for 10 days, after which the fourth leaf of each plant was inoculated with freshly harvested urediospores of CYR31, and samples were collected at 24, 48 and 120 hpi for RNA extraction as described above and histological observation.

### Histological observation of fungal growth

The knockdown wheat leaf samples were collected at 24, 48 and 120 hpi and cut into appropriately sized (1.5–2 mm) segments. The segments were decolorized in ethanol/glacial acetic acid (1:1) for 5–7 days, and the achlorophyllous samples were saturated with chloral hydrate until the leaf tissue became translucent. These transparent leaf segments were examined with a BX‐51 microscope after staining with wheat germ agglutinin conjugated to Alexa‐488 (Invitrogen, Carlsbad, CA, USA) (Ayliffe *et al.*, 2011). At least 50 sites of the stained leaf segments were examined (excitation wavelength 450–490 nm, emission wavelength 515 nm).

## Author Contributions

X.W. and C.T. conceived this research and Q.X. wrote the paper. L.W. and C.Z. constructed the related vectors and cultured the tobacco and wheat plants. L.W. performed RNA extraction and transient expression of proteins. X.W, C.T. and Z.K. revised the manuscript.

## Supporting information


**Fig. S1** Isolating haustoria from wheat leaves infected with *Puccinia striiformis* f. sp.* tritici *(*Pst*)*. *Haustoria were isolated from wheat leaves infected with *Pst* race CYR31 using the concanavalin A (Con A) column method. H, haustoria; C, plant chloroplasts. Bar = 20 μm.

**Fig. S2** Functional analysis of six genes related to metabolism of *Puccinia striiformis* f. sp.* tritici *(*Pst*) by host‐induced gene silencing. (A) The fourth leaves inoculated with urediospores of *Pst* race CYR31 were photographed at 15 days post‐inoculation (dpi). Mock, wheat leaves treated with FES buffer alone. Mild chlorotic mosaic symptoms were observed in wheat inoculated with BSMV: *TaPDS* as a control. (B) The silencing efficiency of metabolism‐related genes in silenced plants. The relative expression of these genes was calculated by the 2^–ΔΔCt^ method. The control (CK) data were from wheat leaves infected with the empty vector (BSMV:γ) at 24, 48 and 120 hours post‐inoculation (hpi). Bars represent mean values ± standard error of three independent sample collections.

**Fig.** **S3** Histological observation of fungal development in knockdown *Pst_11012* plant leaves. A, B and C are represented as the sample BSMV:γ at 24, 48 and 120 hours post‐inoculation (hpi), respectively. D, E and F represent the sample BSMV:*Pst_11012* at 24, 48 and 120 hpi, respectively. SV, substomatal vesicle; HMC, haustorial mother cell; H, haustorium.

**Fig. S4** Immunoblot analysis of Bax protein from *Nicotiana* *benthamiana*. The Bax protein was detected by SDS‐PAGE and western blot with anti‐Bax antibody.

**Fig. S5** The analysis of suppression of PAMP‐triggered immunity. Average number of callose spots per field of view (1 mm^2^) was analysed. The average and standard error form three independent samples of 50 fields each. Buffer control (BC) sample is plant inoculated with water and Dsred as negative controls. Significance was assessed with Student's *t*‐test (asterisk indicates *P* < 0.05).

**Fig. S6** Analysis of transcript level patterns of the other 65 metabolism‐related genes by quantitative reverse transcription PCR. Transcripts were analysed from urediospores and other stages of* Puccinia striiformis* f. sp.* tritici *(*Pst*) infection. Us, urediospores of *Pst*, 6, 12, 24, 36, 48, 72, 96 and 120 hours post‐inoculation (hpi), and 7, 9 and 11 days post‐inoculation (dpi) of wheat leaves. The standard error was obtained from three independent replicates.

**Table S1** RNA‐Seq statistics from germ tubes, urediospores and haustoria.

**Table S2** Differentially expressed genes of haustoria compared with germ tube RNA‐Seq.

**Table S3** Differentially expressed genes of haustoria compared with urediospores RNA‐Seq.

**Table S4** Up‐regulated genes of haustoria compared with germ tube RNA‐Seq and urediospores RNA‐Seq.

**Table S5** Detailed annotations of all genes from *Puccinia striiformis* f. sp. *tritici*.

**Table S6** Haustorial expression of genes.

**Table S7** List of secreted proteins from haustoria RNA‐Seq using SignalP v. 4.1.

**Table S8** Details of screening 14 genes related to *Puccinia striiformis *f. sp.* tritici* metabolism in this study.

**Table S9** Statistical analysis of histological observations from the plant samples of knockdown metabolism‐related genes.

**Table S10** Details of screening effectors of haustoria in this study.

**Table S11** Primers used in this study.

## Data Availability

The data that support the findings of this study are available from the corresponding author on reasonable request.
