# Supplementary material for: Haustoria – arsenals during the interaction between wheat and Puccinia striiformis f. sp. tritici
Source: Mol Plant Pathol. 2019 Nov 27;21(1):83–94. doi: 10.1111/mpp.12882 (PMC6913192; doi:10.1111/mpp.12882)

**Fig. S1.** **Isolating haustoria from infected wheat leaves with *Pst.*** Haustoria were isolated from infected wheat leaves with *Pst* isolate CYR31 using the concanavalin A (Con-A) column. H was represented as haustoria and C was as plant chloroplasts. Bar=20 μm.


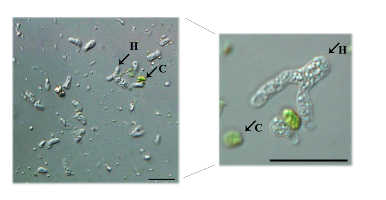

Supplement: Supplementary file 1 — Fig. S1 Isolating haustoria from wheat leaves infected with Puccinia striiformis f. sp. tritici (Pst). Haustoria were isolated from wheat leaves infected with Pst race CYR31 using the concanavalin A (Con A) column method. H, haustoria; C, plant chloroplasts. Bar = 20 μm. [file MPP-21-83-s001.doc]
