# Supplementary material for: Haustoria – arsenals during the interaction between wheat and Puccinia striiformis f. sp. tritici
Source: Mol Plant Pathol. 2019 Nov 27;21(1):83–94. doi: 10.1111/mpp.12882 (PMC6913192; doi:10.1111/mpp.12882)

**Fig. S2. Functional analysis of the other 6 genes related to metabolism of *Pst* by HIGS.** (A) The fourth leaves inoculated with urediniospores of CYR31 were photographed at 15 dpi. Mock, wheat leaves treated with FES buffer alone. Mild chlorotic mosaic symptoms were observed in wheat inoculated with BSMV: *TaPDS* as a control. (B) The silencing efficiency of metabolism-related genes in silenced plants. The relative expression of these genes was calculated using the 2^–ΔΔCT^ method. The CK data were from wheat leaves infected with the empty vector (BSMV: γ) at 24hpi, 48hpi and 120hpi. Bars represent mean values ± standard error of three independent sample collections.


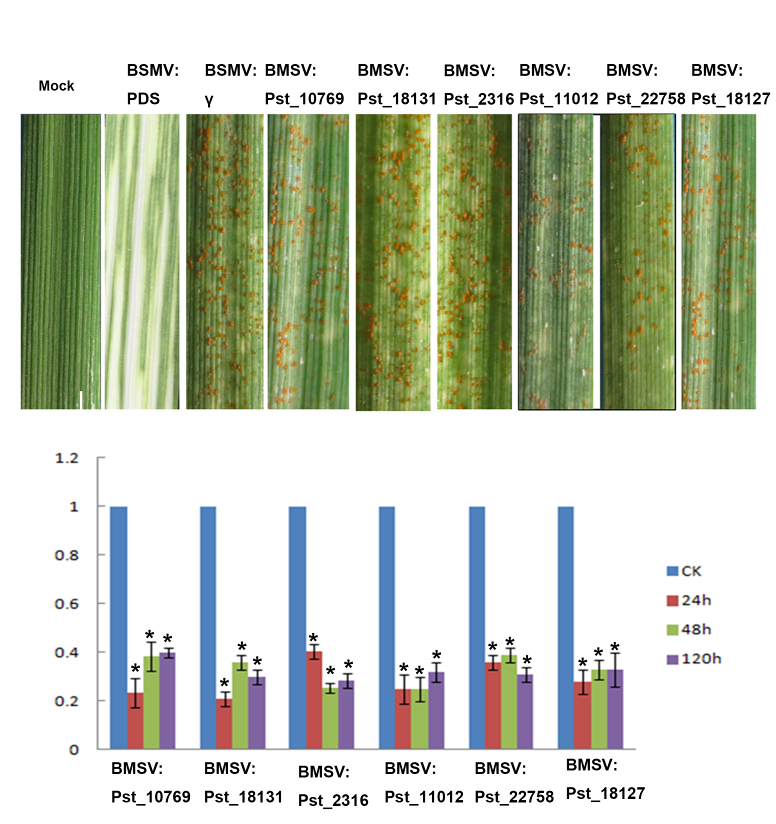

Supplement: Supplementary file 2 — Fig. S2 Functional analysis of six genes related to metabolism of Puccinia striiformis f. sp. tritici (Pst) by host‐induced gene silencing. (A) The fourth leaves inoculated with urediospores of Pst race CYR31 were photographed at 15 days post‐inoculation (dpi). Mock, wheat leaves treated with FES buffer alone. Mild chlorotic mosaic symptoms were observed in wheat inoculated with BSMV: TaPDS as a control. (B) The silencing efficiency of metabolism‐related genes in silenced plants. The relative expression of these genes was calculated by the 2–ΔΔCt method. The control (CK) data were from wheat leaves infected with the empty vector (BSMV:γ) at 24, 48 and 120 hours post‐inoculation (hpi). Bars represent mean values ± standard error of three independent sample collections. [file MPP-21-83-s002.doc]
