# Supplementary material for: Haustoria – arsenals during the interaction between wheat and Puccinia striiformis f. sp. tritici
Source: Mol Plant Pathol. 2019 Nov 27;21(1):83–94. doi: 10.1111/mpp.12882 (PMC6913192; doi:10.1111/mpp.12882)
Supplement: Supplementary file 3 — Fig. S3 Histological observation of fungal development in knockdown Pst_11012 plant leaves. A, B and C are represented as the sample BSMV:γ at 24, 48 and 120 hours post‐inoculation (hpi), respectively. D, E and F represent the sample BSMV:Pst_11012 at 24, 48 and 120 hpi, respectively. SV, substomatal vesicle; HMC, haustorial mother cell; H, haustorium. [file MPP-21-83-s003.doc]

**Fig. S3. Histological observation of fungal development in knock-down *Pst_11012* plant leaves.** A, B and C were represented as the sample BSMV: γ at 24 hpi, 48 hpi and 120 hpi, respectively. D, E and F were represented as the sample BSMV: *Pst_11012* at 24 hpi, 48 hpi and 120 hpi. SV, substomatal vesicle; HMC, haustorial mother Cell; H, haustorium.

**
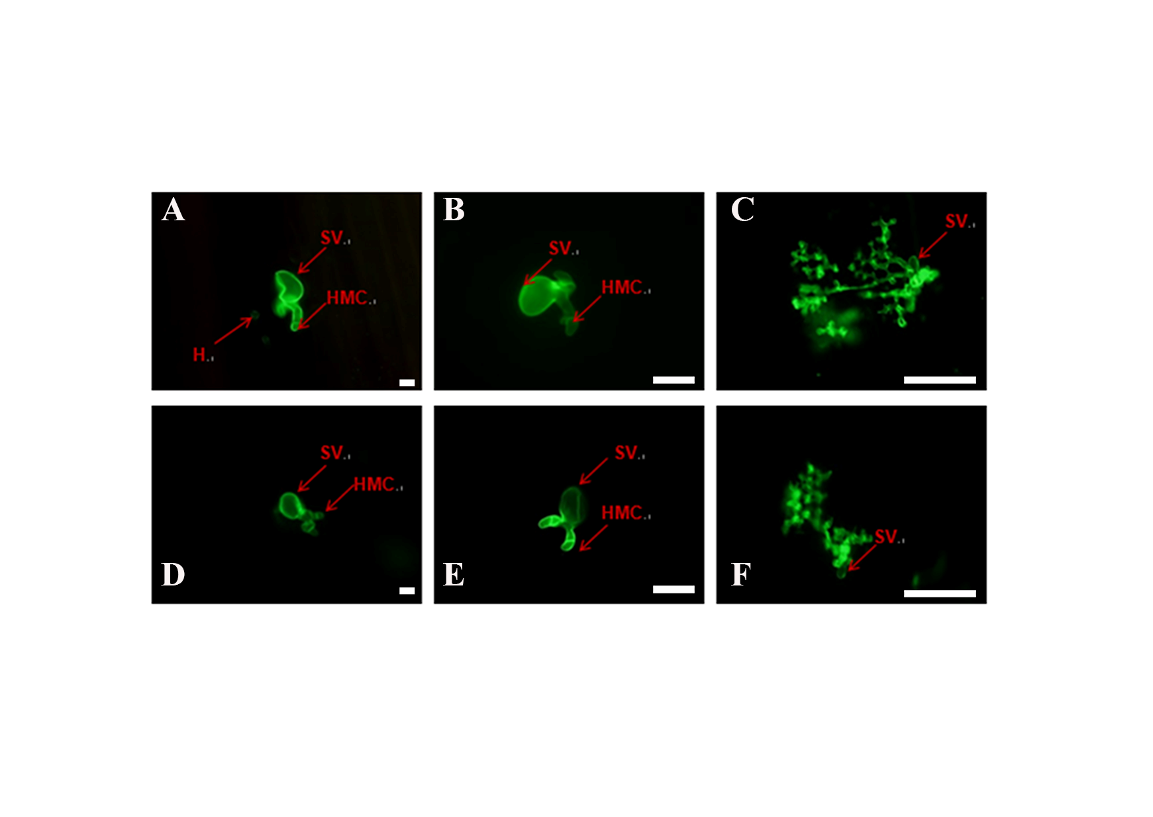
**
