# Supplementary material for: Haustoria – arsenals during the interaction between wheat and Puccinia striiformis f. sp. tritici
Source: Mol Plant Pathol. 2019 Nov 27;21(1):83–94. doi: 10.1111/mpp.12882 (PMC6913192; doi:10.1111/mpp.12882)

Fig. S4. Immunoblot analysis of Bax protein from N. *benthamiana*. The Bax protein was detected by SDS-PAGE and western blot with anti-Bax antibody.


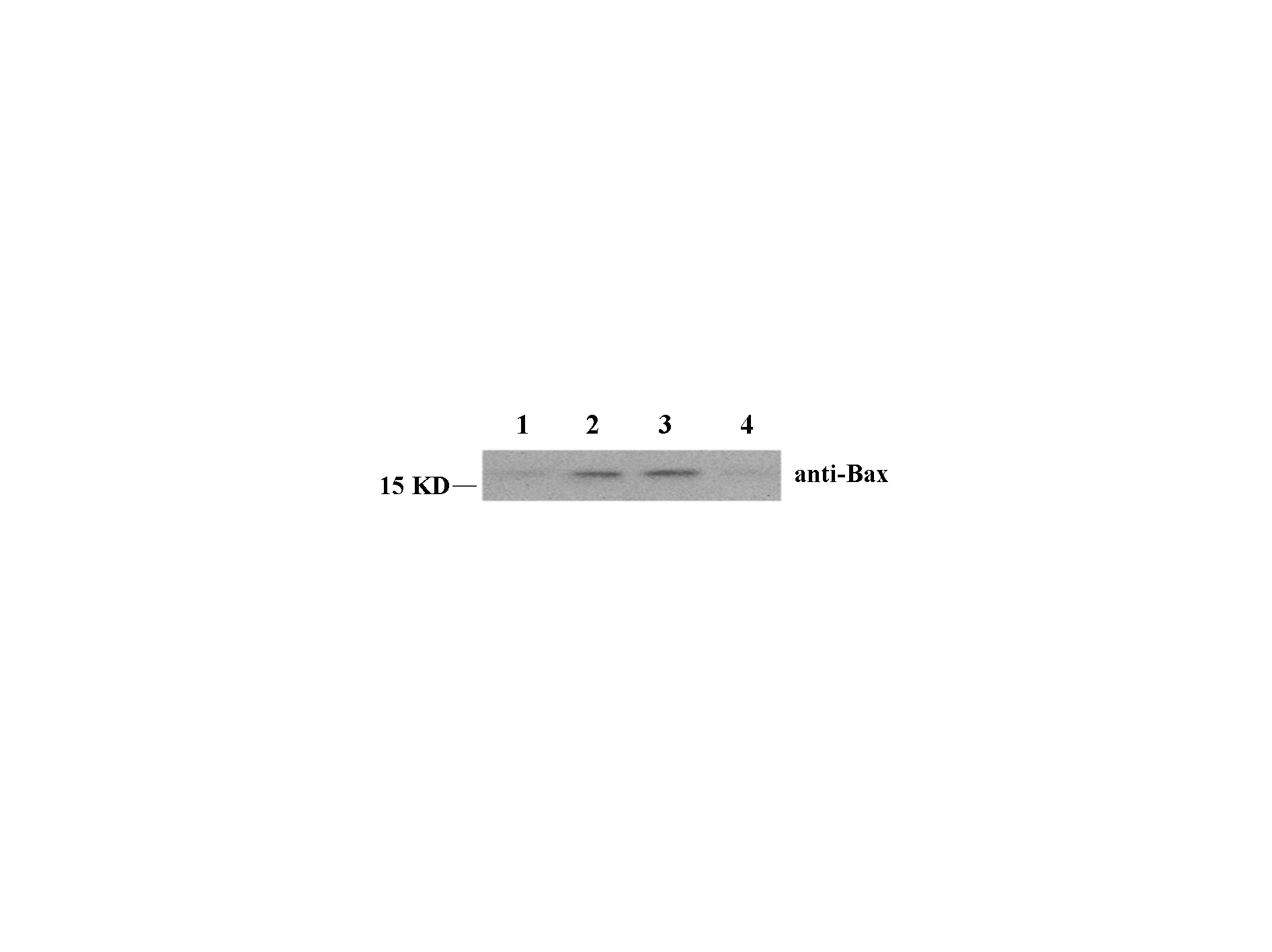

Supplement: Supplementary file 4 — Fig. S4 Immunoblot analysis of Bax protein from Nicotiana benthamiana. The Bax protein was detected by SDS‐PAGE and western blot with anti‐Bax antibody. [file MPP-21-83-s004.doc]
