# Supplementary material for: Haustoria – arsenals during the interaction between wheat and Puccinia striiformis f. sp. tritici
Source: Mol Plant Pathol. 2019 Nov 27;21(1):83–94. doi: 10.1111/mpp.12882 (PMC6913192; doi:10.1111/mpp.12882)

**Fig. S5. The analysis of suppression of plant PTI.** Average number of callose spots per fields of view (1mm^2^) were analyzed. the avereage and standard error were form three independents samples with fifty fields. BC sample was plant inoculated with water and Dsred acts as a negative control. A signifcance were assessed with a student's t-test (Asterisk presents P<0.05).


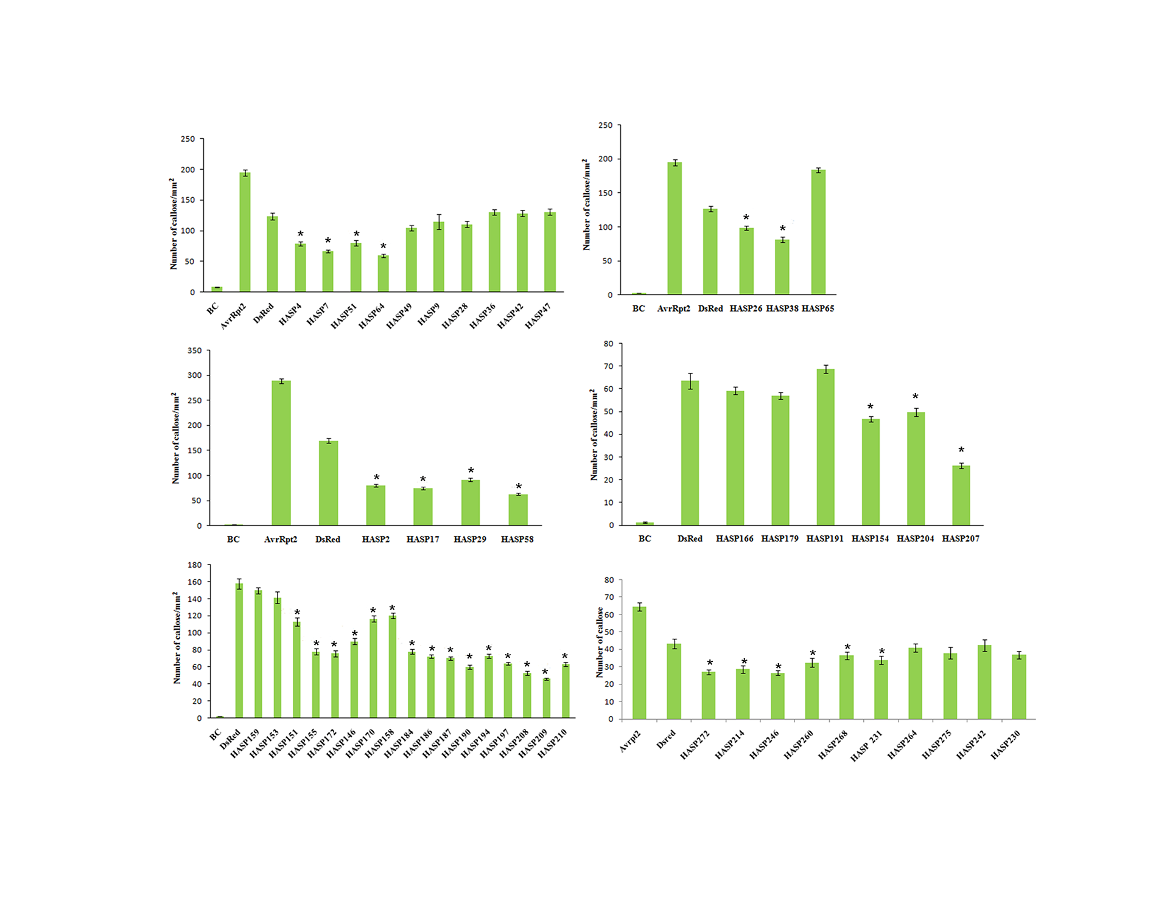

Supplement: Supplementary file 5 — Fig. S5 The analysis of suppression of PAMP‐triggered immunity. Average number of callose spots per field of view (1 mm2) was analysed. The average and standard error form three independent samples of 50 fields each. Buffer control (BC) sample is plant inoculated with water and Dsred as negative controls. Significance was assessed with Student's t‐test (asterisk indicates P < 0.05). [file MPP-21-83-s005.doc]
