# Supplementary material for: Haustoria – arsenals during the interaction between wheat and Puccinia striiformis f. sp. tritici
Source: Mol Plant Pathol. 2019 Nov 27;21(1):83–94. doi: 10.1111/mpp.12882 (PMC6913192; doi:10.1111/mpp.12882)

**Fig. S6. Transcript level patterns analysis of the other 65 metabolism related genes by RT-PCR.** Transcripts were analyzed from urediniospores and other stages of *Pst* infection. Us, urediniospores of *Pst*, 6,12,24,36,48,72,96 and 120 h, hours post-inculation of wheat leaves. 7, 9 and 11 dpi, day post-inoculation of wheat leaves. The standard error was obtained from three independent replicates.


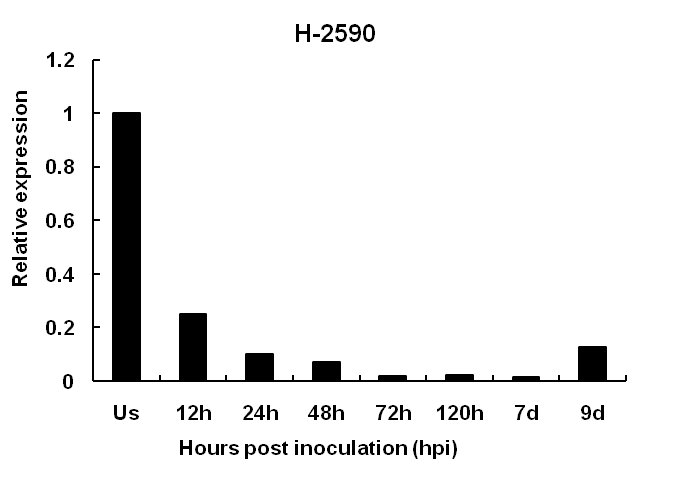

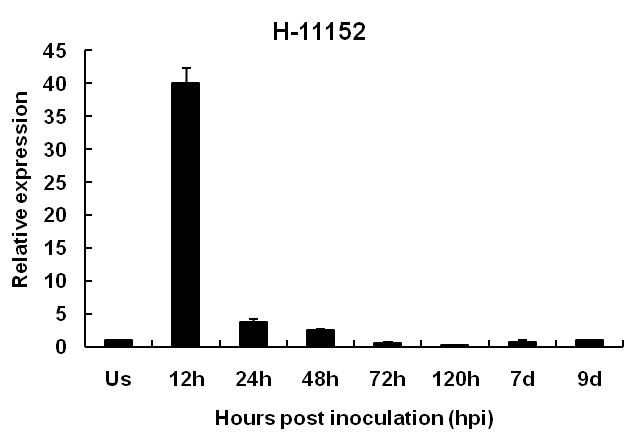

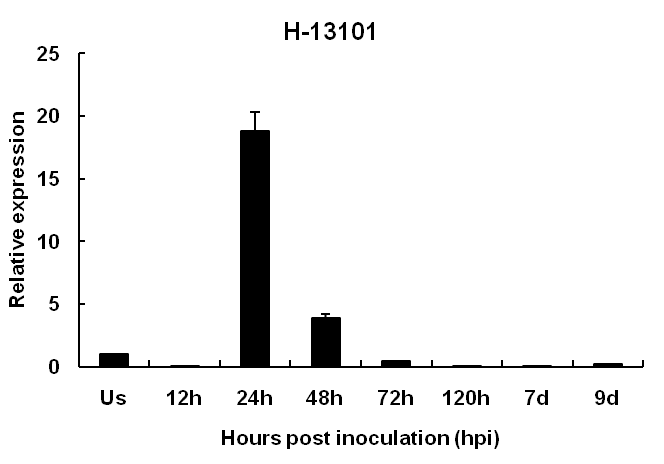

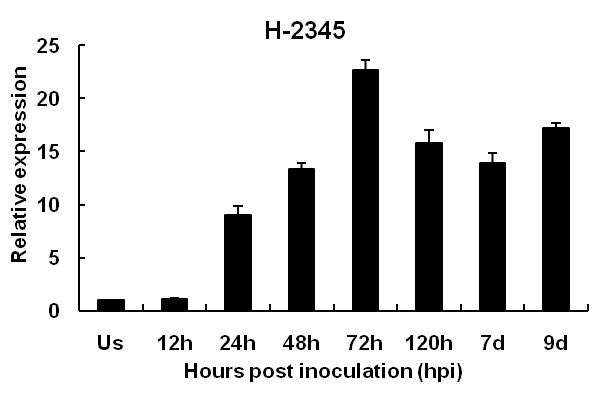

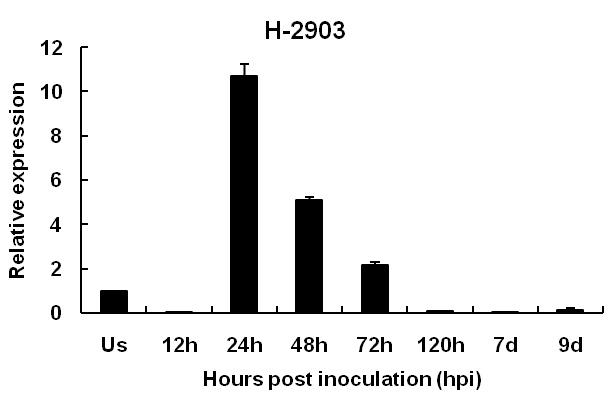


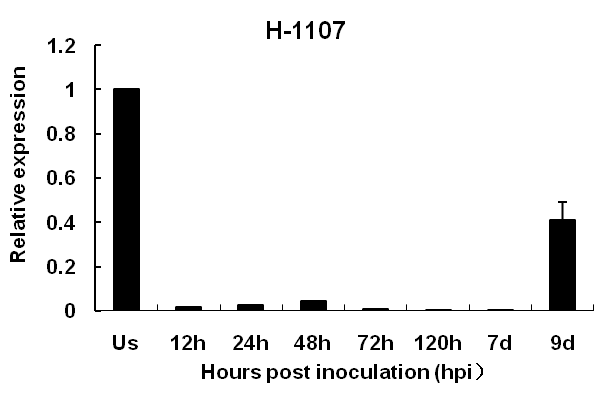

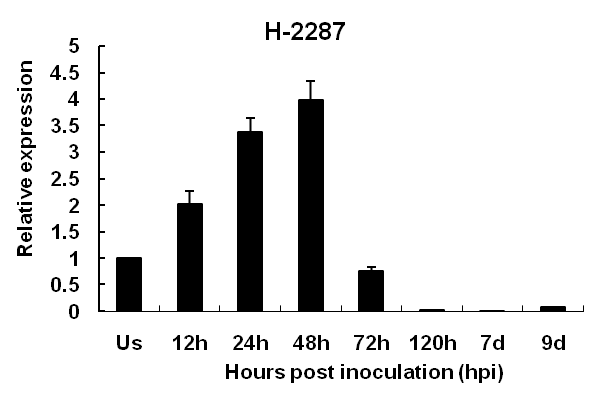


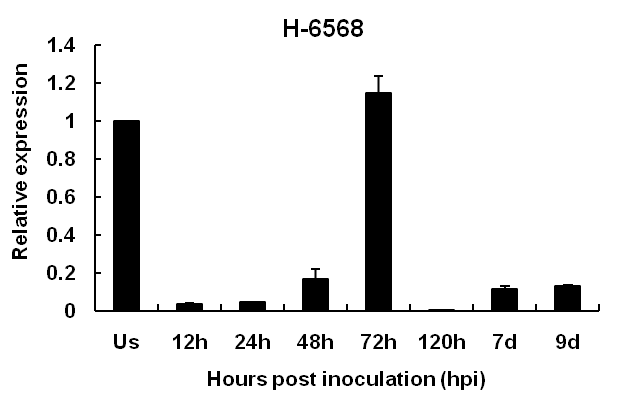

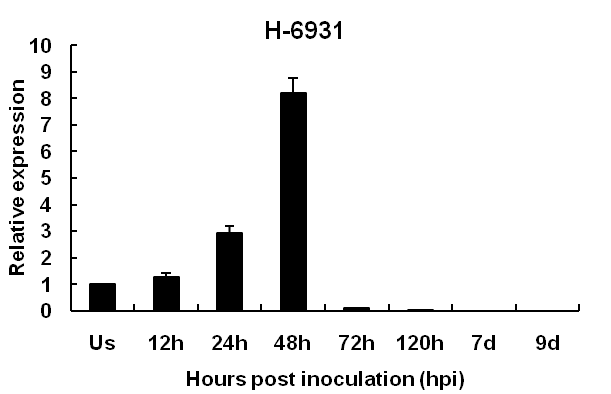


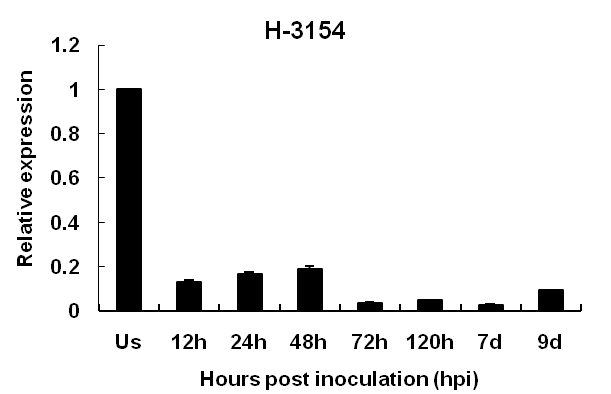

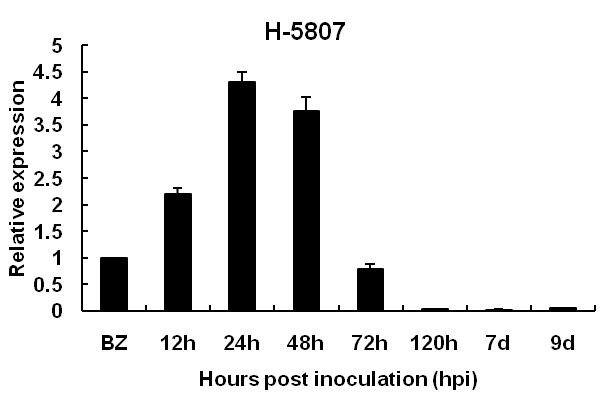

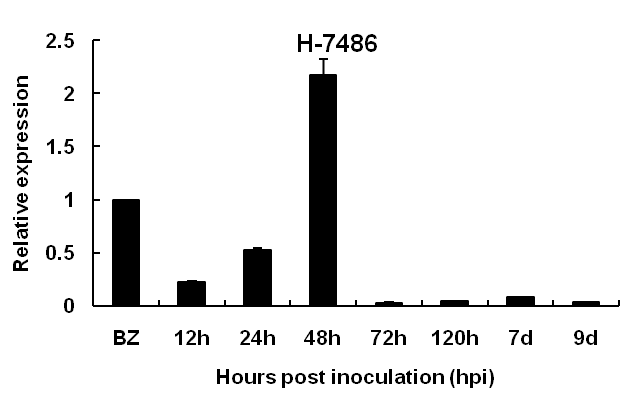

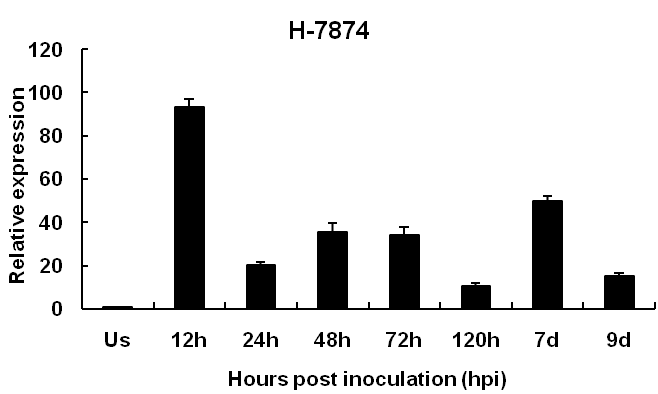

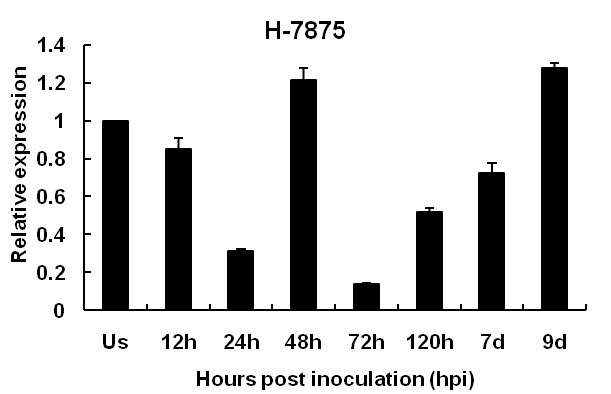

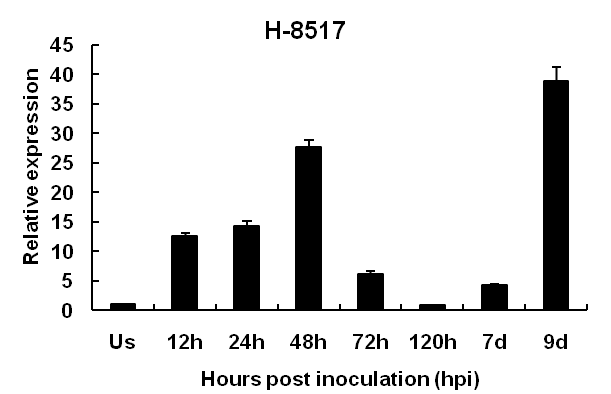


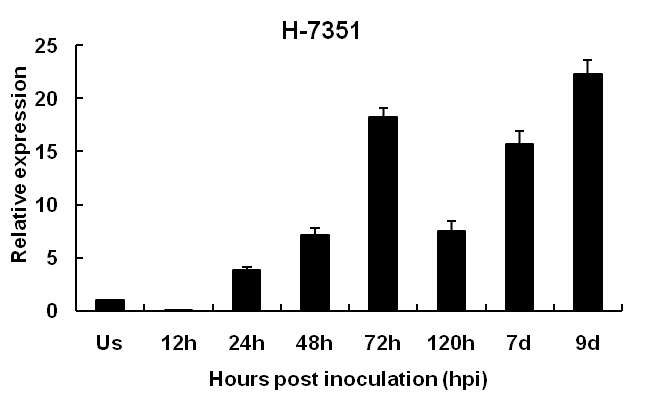

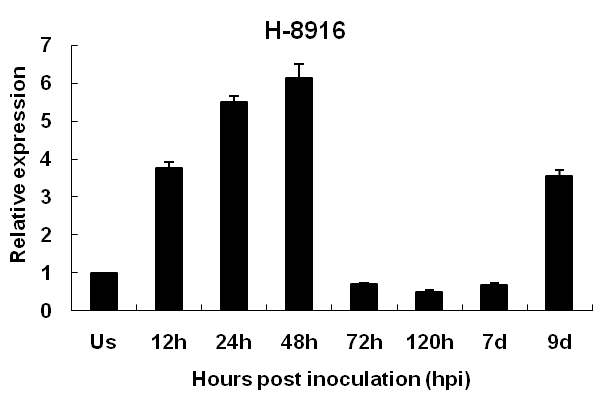

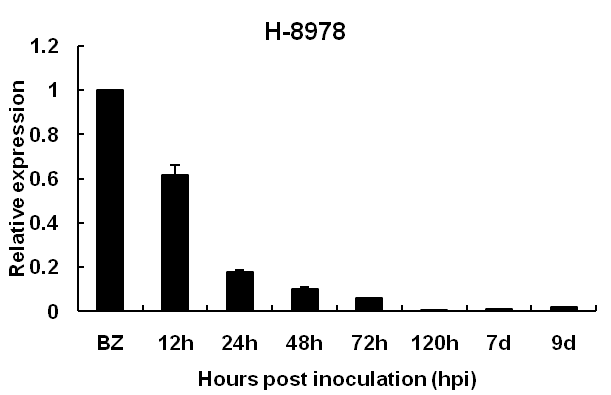

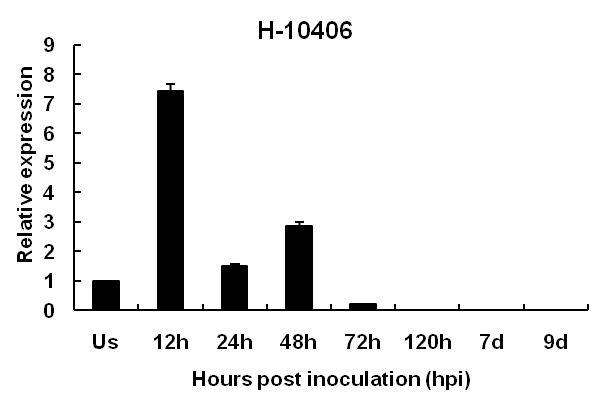

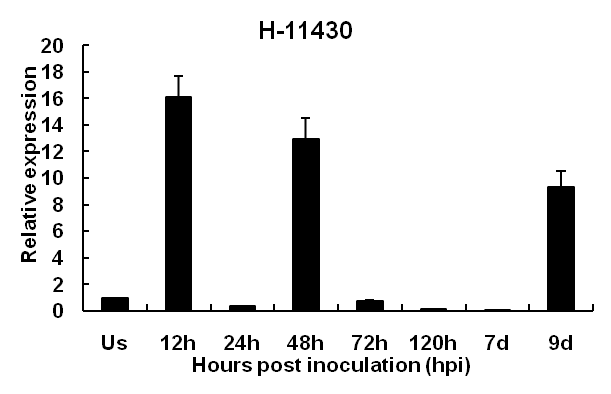

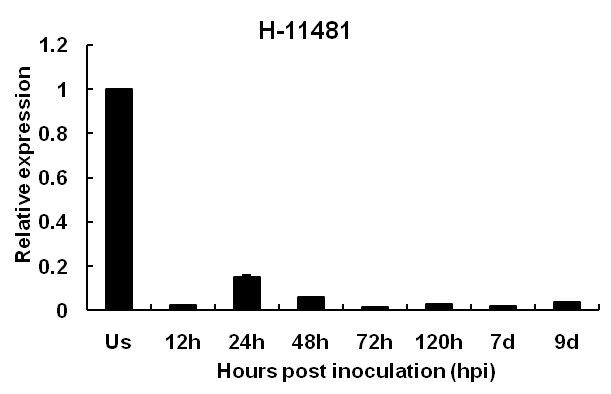

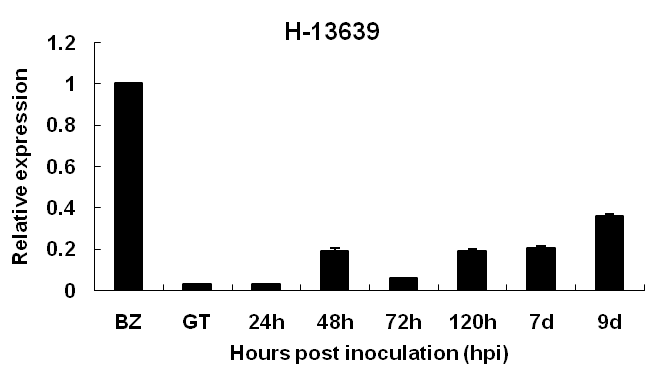

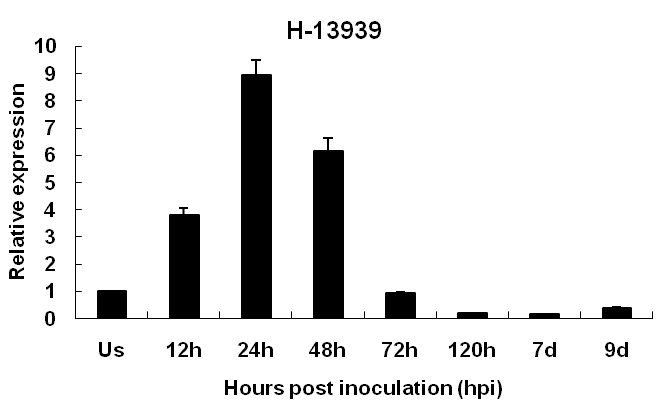

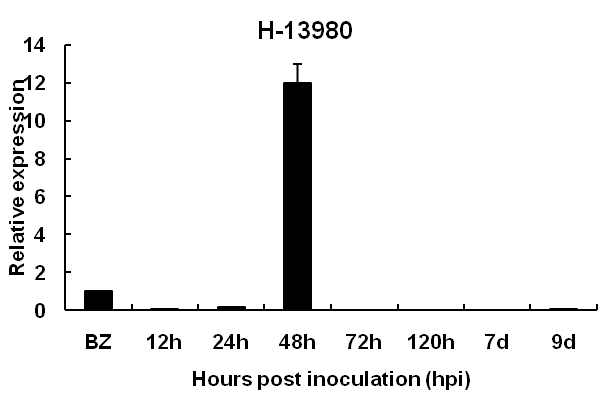


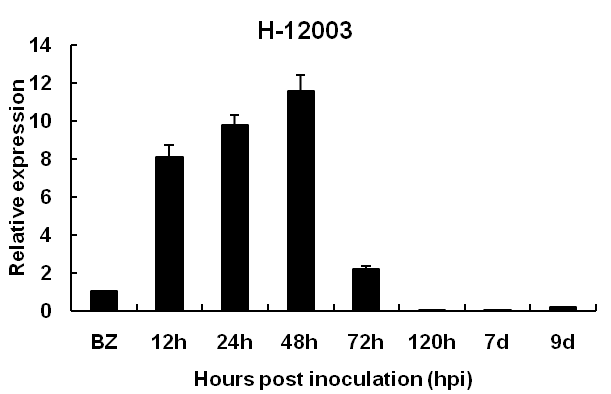

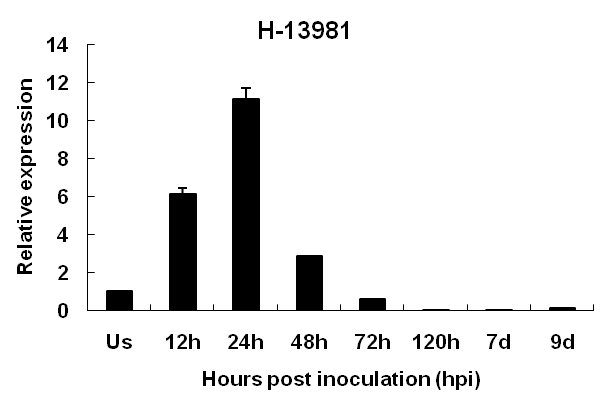


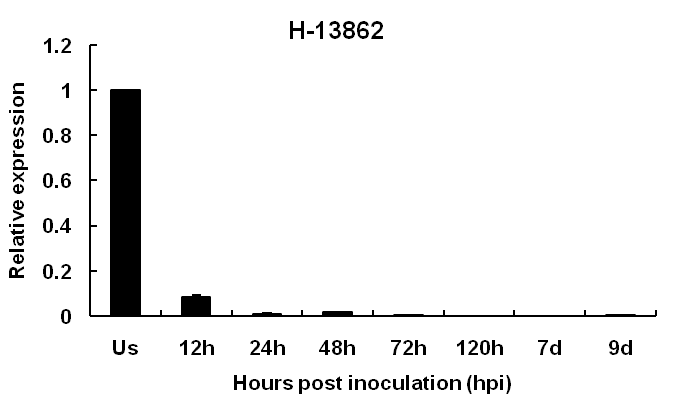


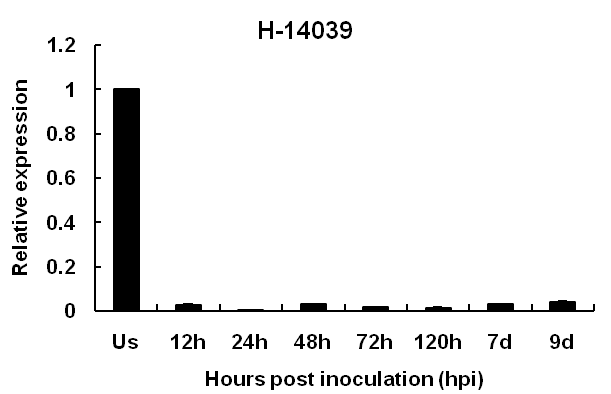

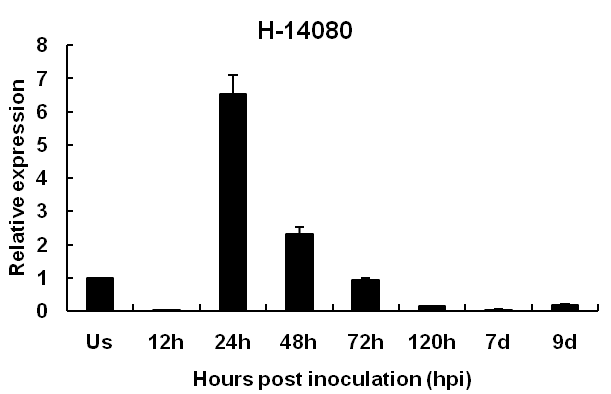

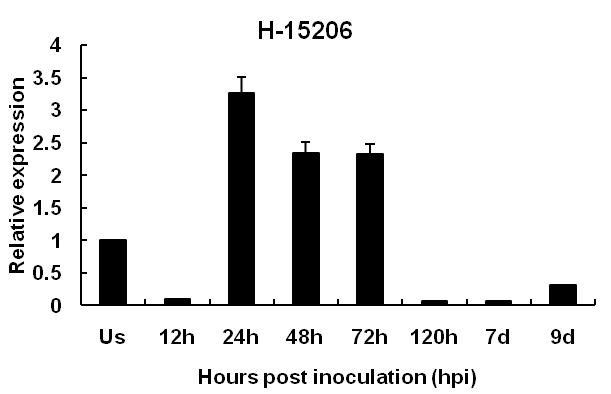


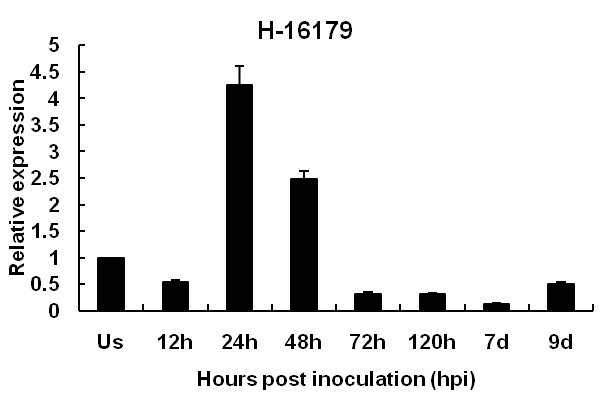

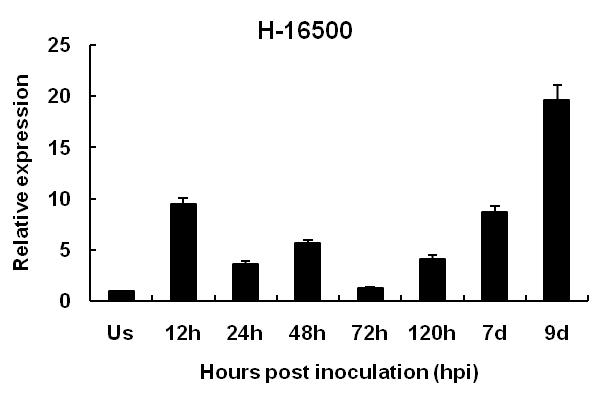

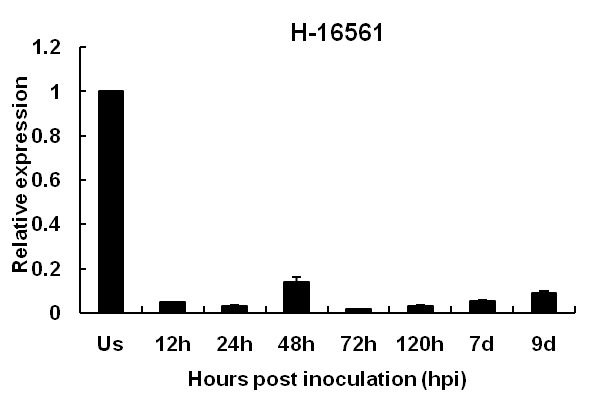

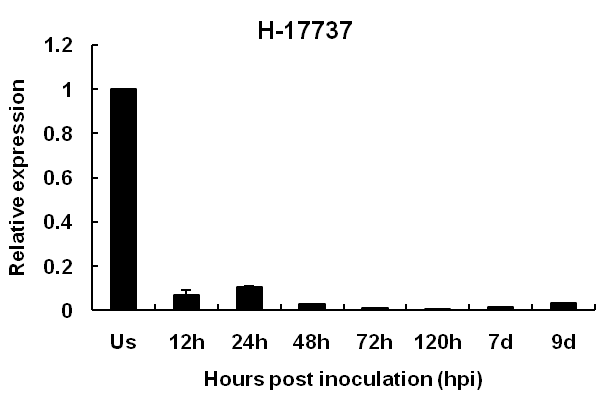

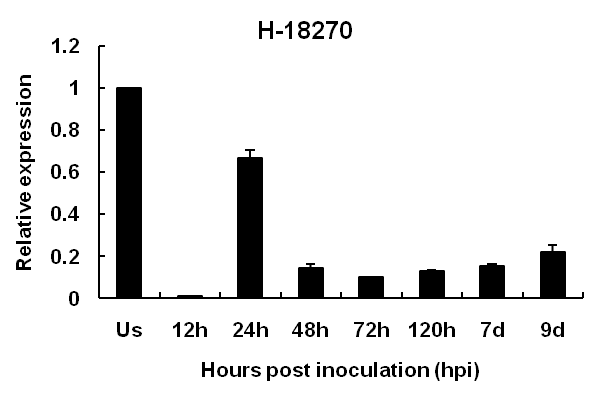


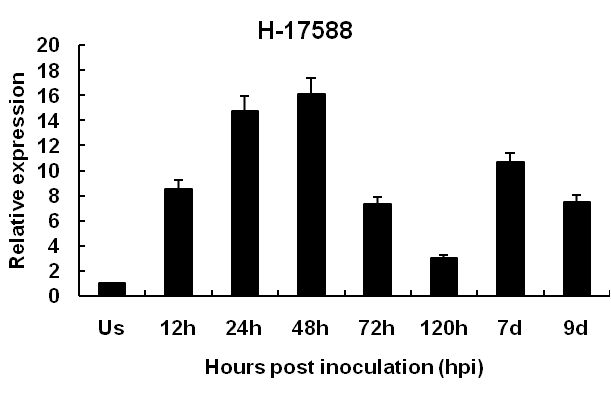

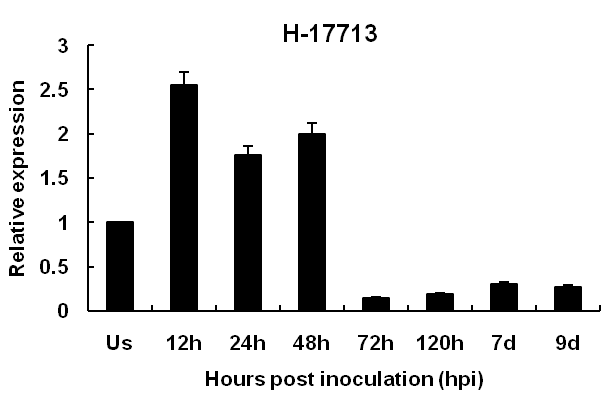


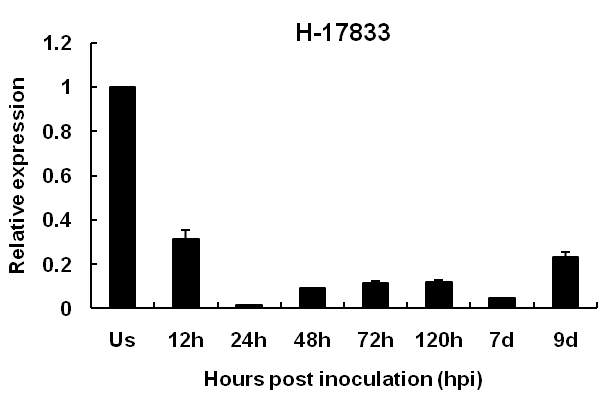

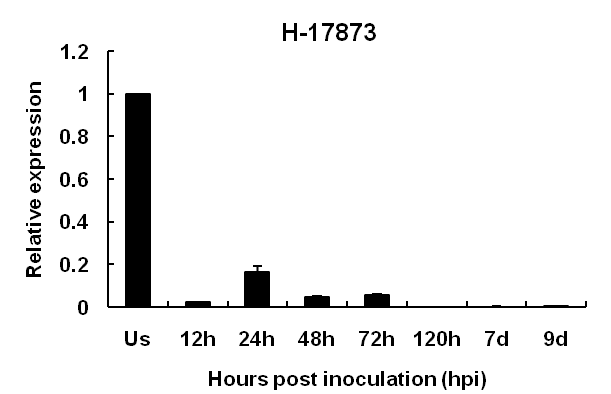


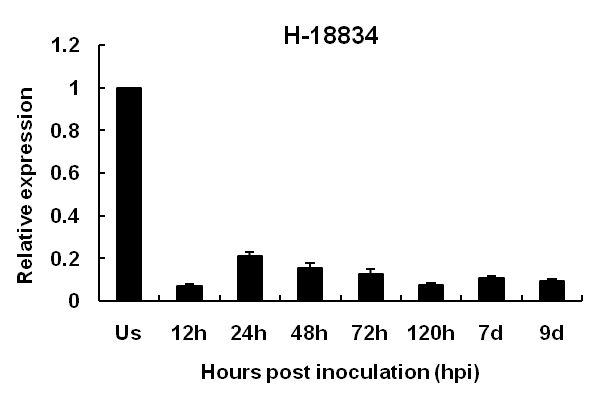

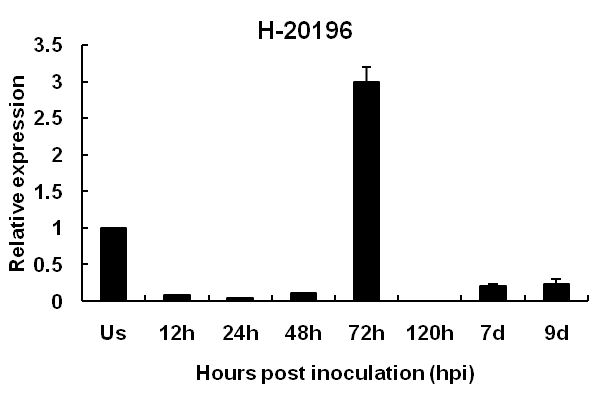

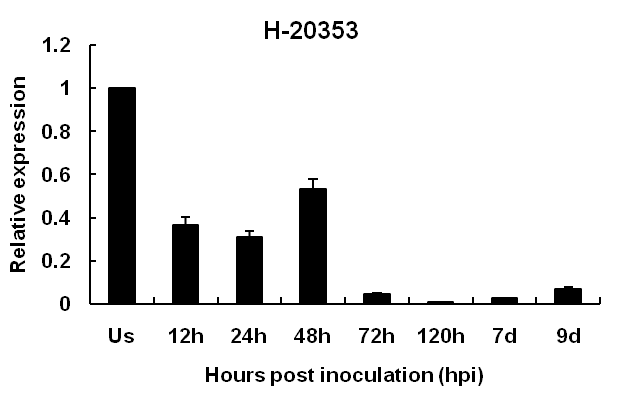


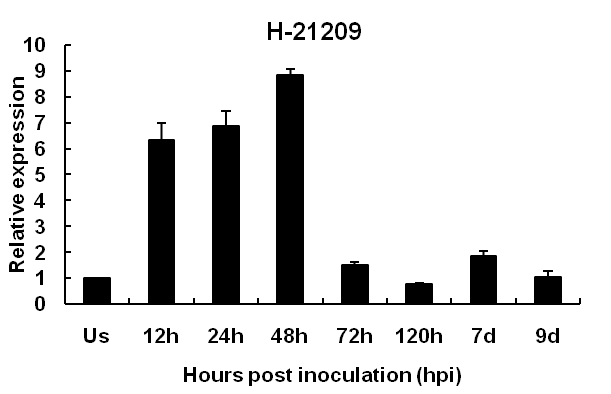

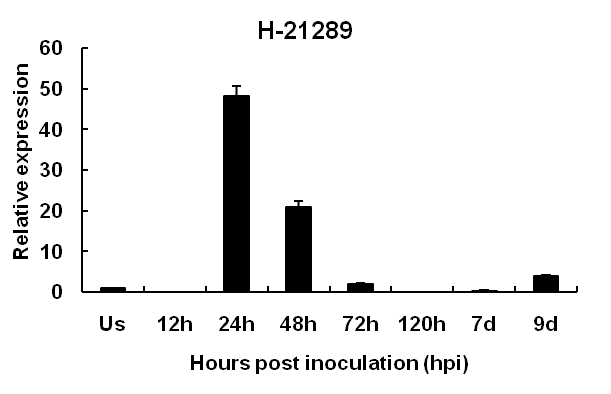

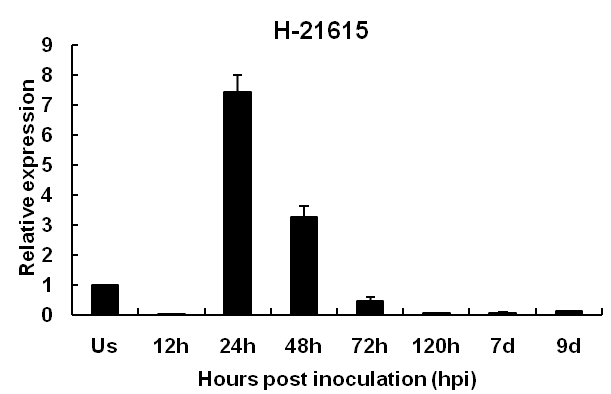


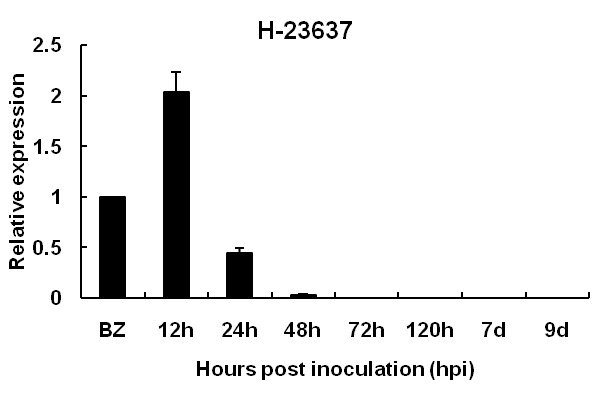

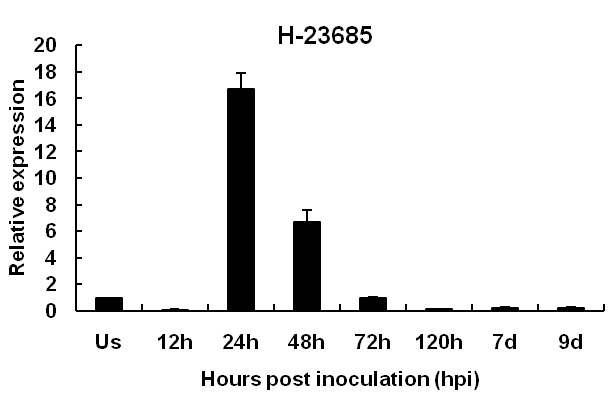

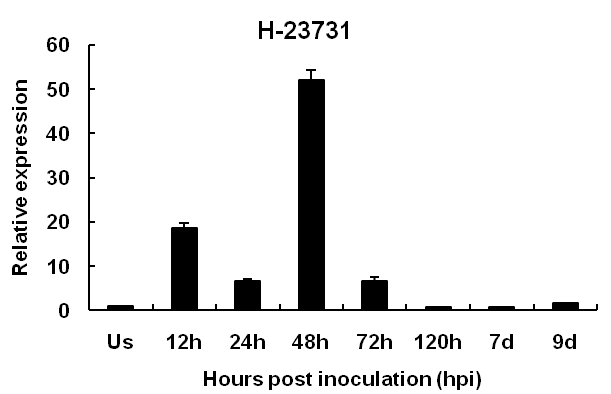

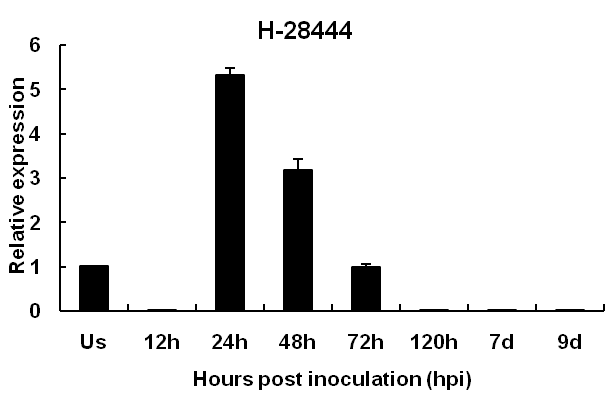


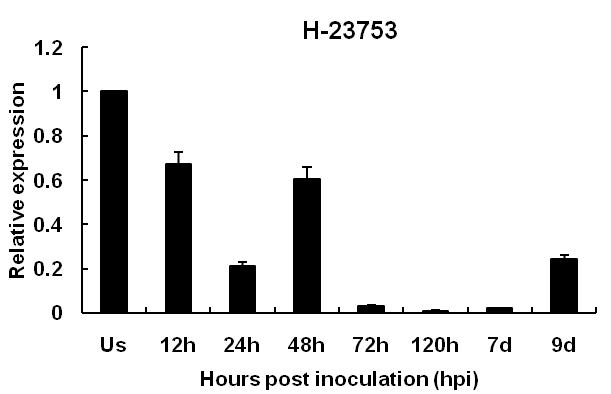

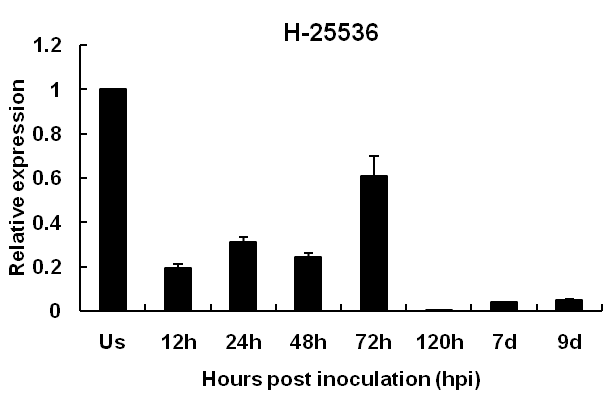

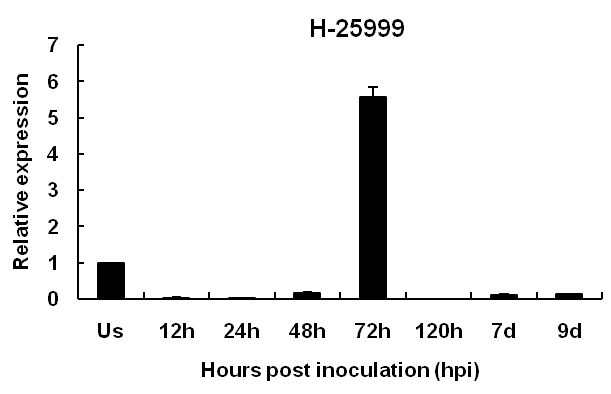

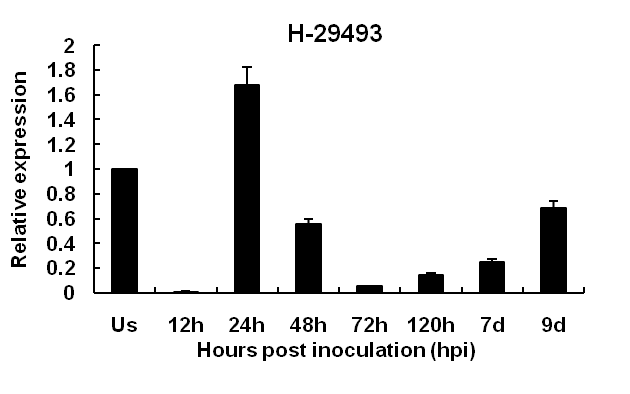


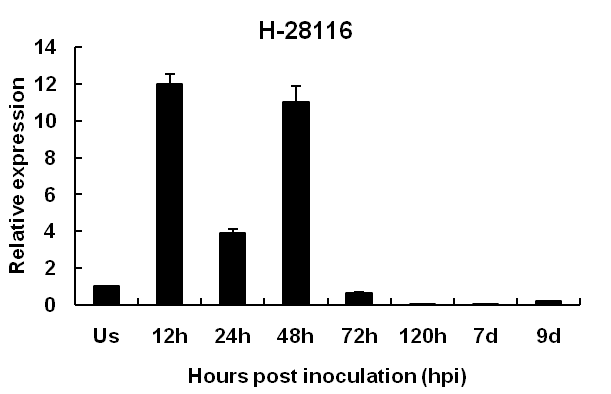

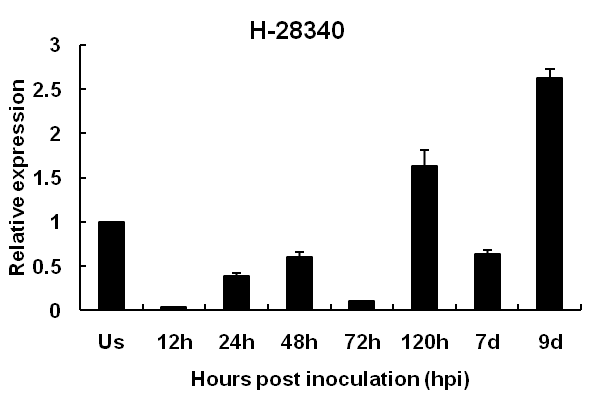


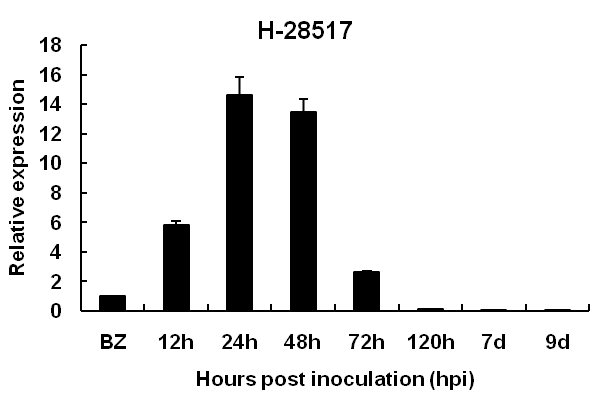

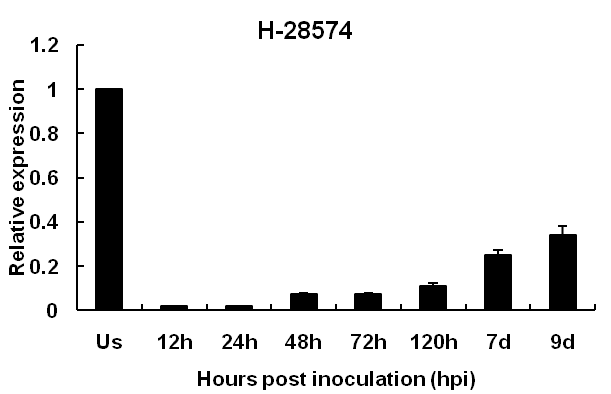

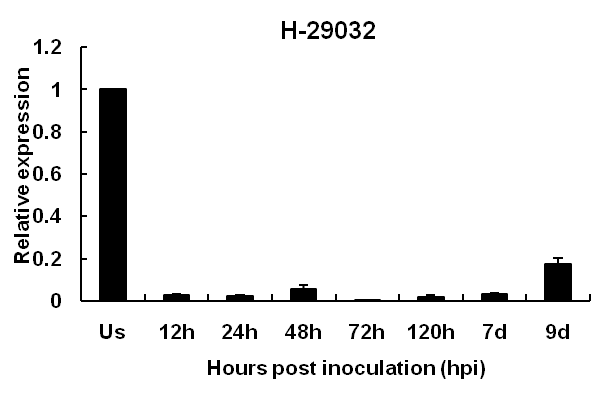


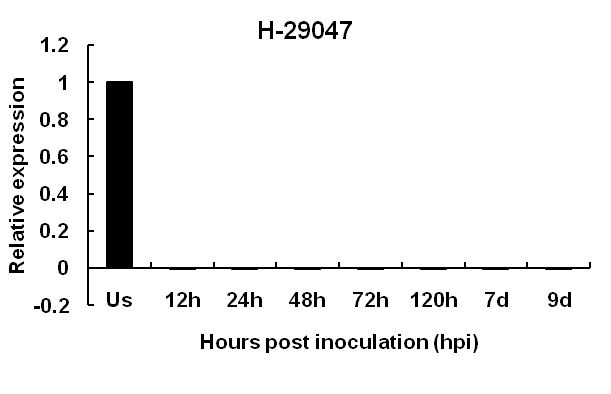

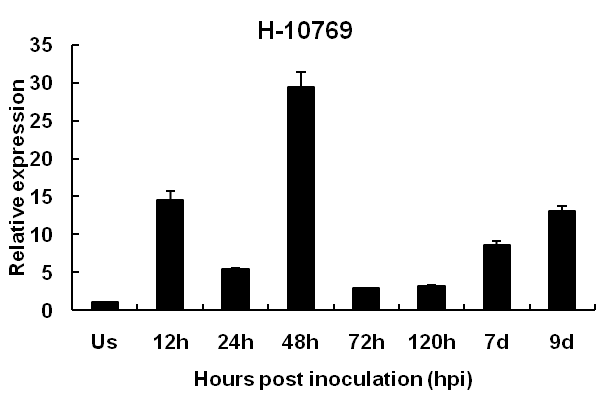


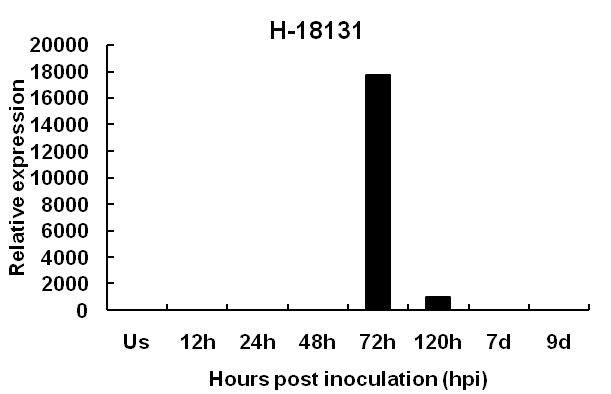

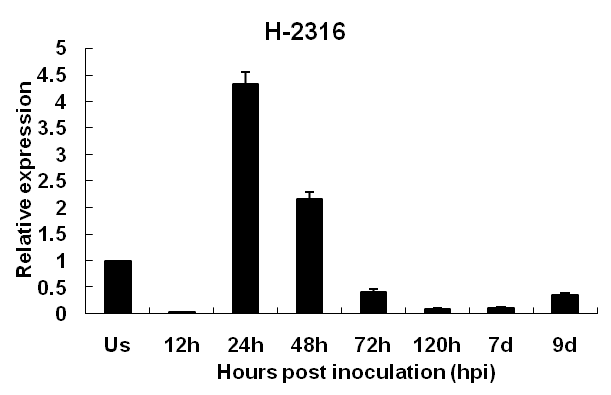

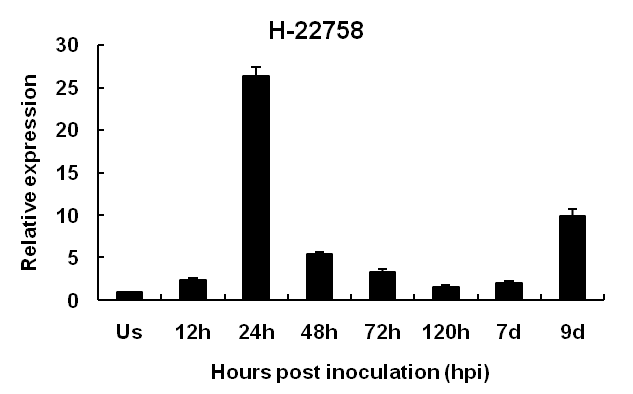


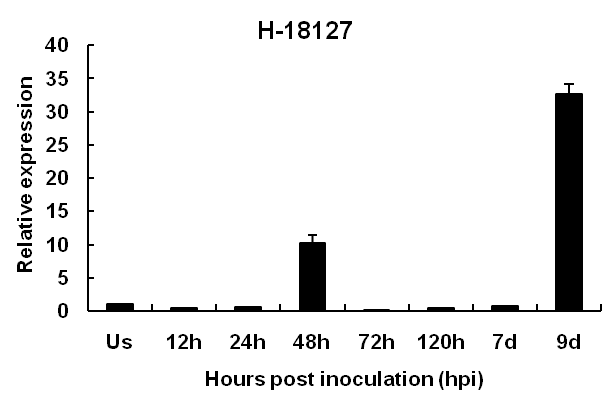

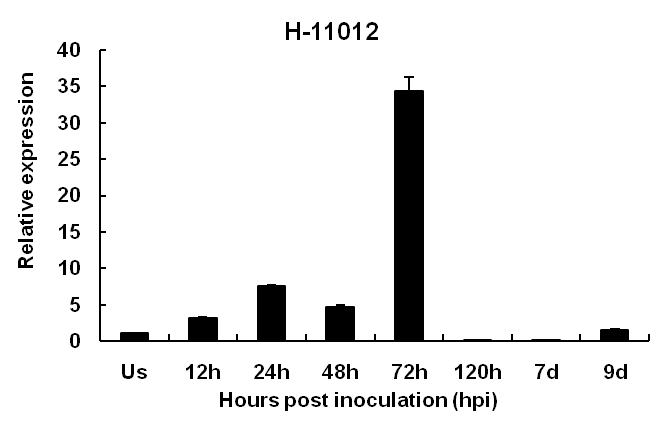

Supplement: Supplementary file 6 — Fig. S6 Analysis of transcript level patterns of the other 65 metabolism‐related genes by quantitative reverse transcription PCR. Transcripts were analysed from urediospores and other stages of Puccinia striiformis f. sp. tritici (Pst) infection. Us, urediospores of Pst, 6, 12, 24, 36, 48, 72, 96 and 120 hours post‐inoculation (hpi), and 7, 9 and 11 days post‐inoculation (dpi) of wheat leaves. The standard error was obtained from three independent replicates. [file MPP-21-83-s006.doc]
